# An updated advancement of bifunctional IL-27 in inflammatory autoimmune diseases

**DOI:** 10.3389/fimmu.2024.1366377

**Published:** 2024-03-19

**Authors:** Wang-Dong Xu, Da-Cheng Wang, Ming Zhao, An-Fang Huang

**Affiliations:** ^1^ Department of Evidence-Based Medicine, School of Public Health, Southwest Medical University, Luzhou, Sichuan, China; ^2^ Institute of Dermatology, Chinese Academy of Medical Sciences and Peking Union Medical College, Nanjing, China; ^3^ Key Laboratory of Basic and Translational Research on Immune-Mediated Skin Diseases, Chinese Academy of Medical Sciences, Nanjing, China; ^4^ Department of Dermatology, Hunan Key Laboratory of Medical Epigenomics, Second Xiangya Hospital, Central South University, Changsha, China; ^5^ Department of Rheumatology and Immunology, Affiliated Hospital, Southwest Medical University, Luzhou, Sichuan, China

**Keywords:** IL-27, autoimmunity, immune cell, function, immunity

## Abstract

Interleukin-27 (IL-27) is a member of the IL-12 family. The gene encoding IL-27 is located at chromosome 16p11. IL-27 is considered as a heterodimeric cytokine, which consists of Epstein–Barr virus (EBV)-induced gene 3 (Ebi3) and IL-27p28. Based on the function of IL-27, it binds to receptor IL-27rα or gp130 and then regulates downstream cascade. To date, findings show that the expression of IL-27 is abnormal in different inflammatory autoimmune diseases (including systemic lupus erythematosus, rheumatoid arthritis, Sjogren syndrome, Behcet’s disease, inflammatory bowel disease, multiple sclerosis, systemic sclerosis, type 1 diabetes, Vogt–Koyanagi–Harada, and ankylosing spondylitis). Moreover, *in vivo* and *in vitro* studies demonstrated that IL-27 is significantly in3volved in the development of these diseases by regulating innate and adaptive immune responses, playing either an anti-inflammatory or a pro-inflammatory role. In this review, we comprehensively summarized information about IL-27 and autoimmunity based on available evidence. It is hoped that targeting IL-27 will hold great promise in the treatment of inflammatory autoimmune disorders in the future.

## 1 Introduction

Interleukin-27 (IL-27) is a heterodimeric member of the IL-12 cytokine family. IL-27 has two subunits, Epstein–Barr virus (EBV)-induced gene 3 (Ebi3) and IL-27p28. The IL-27 receptor complex consists of IL-27rα and glycoprotein 130 (gp130). IL-27 and its receptor are expressed in some non-immune cells, such as renal tubular epithelial cells (1) and cardiac Sca-1^+^ cells (2), and some immune cells, such as monocytes, macrophages, dendritic cells (DCs), neutrophils, T cells, and B cells. IL-27 binds to receptors and then regulates downstream signaling such as mitogen-activated protein kinase (MAPK), nuclear factor-kappa B (NF-κB), and signal transducer and activator of transcriptions (STATs), performing a significant role in innate and adaptive immune response. To date, much information has pointed out bifunctional IL-27 in inflammatory responses (both pro-inflammatory and anti-inflammatory effects). On the one hand, the pro-inflammatory effects of IL-27 were discussed in DCs and different effector T helper (Th) cells. On the other hand, IL-27 suppressed proliferation, apoptosis of monocytes, macrophages, DCs, and B cells. Moreover, evidence based on the association of IL-27 with inflammatory autoimmune diseases indicated that IL-27 not only inhibits autoimmunity development but also promotes autoimmune disease pathogenesis. Therefore, in this review, we updated current knowledge about bifunctional IL-27 in inflammatory autoimmune disorders through regulating different immune cells. It is hoped that, in the future, more precise methods to targeting abnormally expressed IL-27 will bring potential for inhibiting autoimmune disease pathogenesis.

## 2 Role of IL-27 in innate immunity

### 2.1 IL-27 mainly promotes the pro-inflammatory activity of monocytes

**Figure 1:**
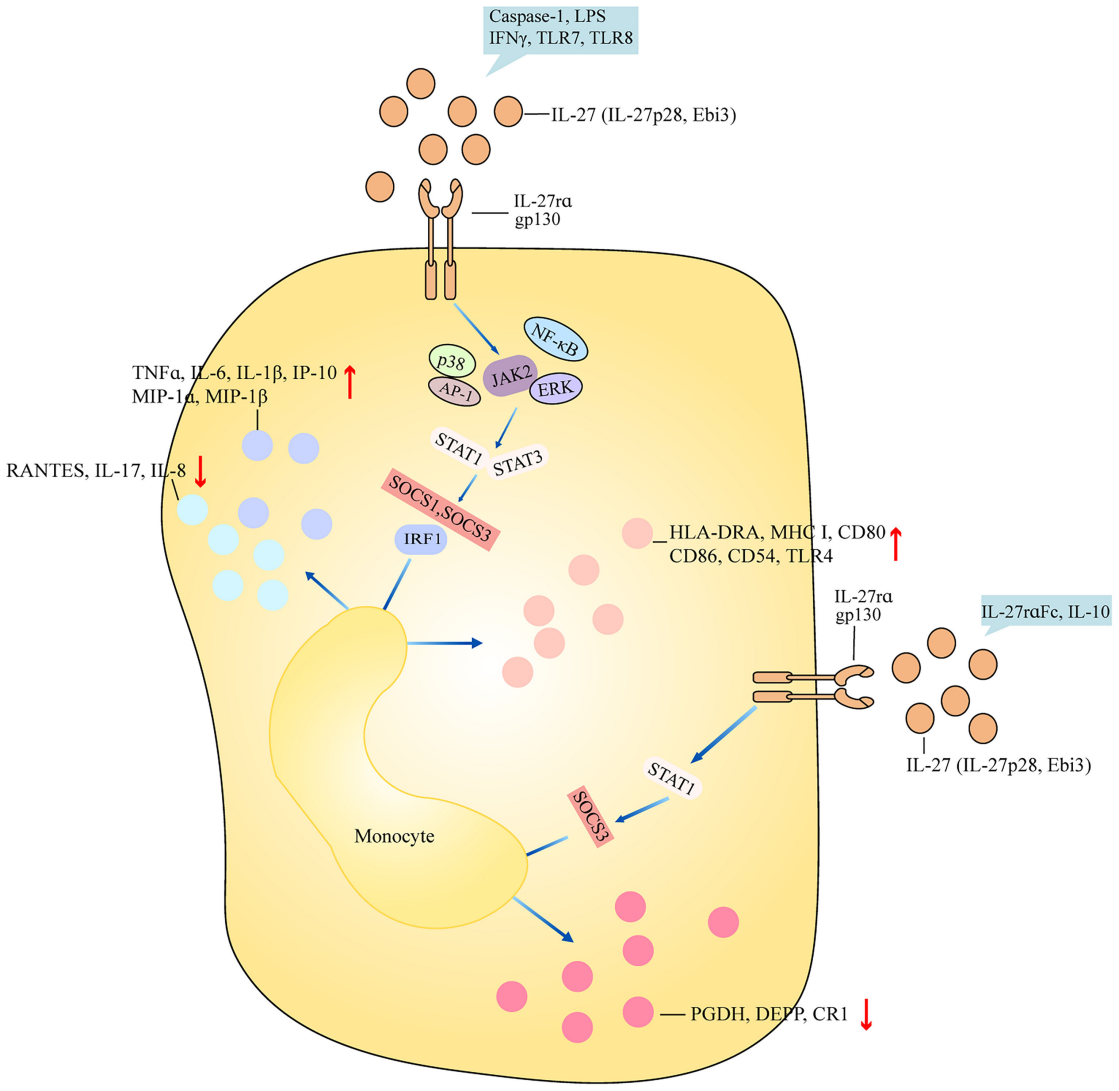
Role of IL-27 in monocyte. IL-27 (IL-27p28, Ebi3) binds to receptor IL-27rα and gp130 and activates downstream signaling including ERK, NF-κB, AP-1, and JAK2, and then regulates activation of cascades STAT1/STAT3 and SOCS1/SOCS3/IRF1. Finally, after the production of inflammatory components, surface markers are differently regulated by bifunctional IL-27. IL-27 promotes the production of inflammatory cytokines such as TNFα, IL-6, and IP-10 and surface markers such as HLA-DRA, CD80, and CD86 in monocytes after caspse-1, LPS, IFNγ, TLR7, and TLR8 stimulation. Stimulation of IL-27-treated monocytes with IL-27rα and IL-10 downregulates expression of PGDH, DEPP, and CR1 by STAT1 and SOCS3. Ebi3, Epstein–Barr virus (EBV)-induced gene 3; ERK, extracellular regulated protein kinases; NF-κB, nuclear factor-kappa B; AP-1, activator protein-1; JAK2, janus kinase 2; STAT1, signal transducer and activator of transcription 1; SOCS1, suppressor of cytokine signaling 1; IRF1, interferon regulatory factor 1; TNFα, tumor necrosis factor alpha; IP-10, interferon γ (IFNγ)-inducible protein 10; HLA-DRA, human leukocyte antigen-DR alpha; LPS, lipopolysaccharide; TLR7, toll-like receptor 7; PGDH, prostaglandin dehydrogenase; DEPP, decidual protein-induced progesterone; CR1, complement receptor 1.

Monocytes are a kind of innate immune cells with diverse functions ranging from phagocytosis of microorganisms to forming a bridge with adaptive immunity and play an important role in inflammation. THP-1 cell is a cell line that was developed by Tsuchiya and colleagues in 1980 from a 1-year-old male patient’s peripheral blood (3). This patient suffered from acute monocytic leukemia. THP-1 cells can resemble the morphological and differentiation properties of primary monocytes (3). THP-1 cells stimulated with IL-27 showed increased expression of interferon γ (IFNγ)-inducible protein 10 (IP-10) and human leukocyte antigen-DR alpha (HLA-DRA) and surface expression of MHC class I, CD80, CD86, CD54, and types III and IV CIITA (4). IL-27-treated THP-1 cells had elevated expression of toll-like receptor 4 (TLR4), CD14, tumor necrosis factor alpha (TNFα), IL-6, and IL-8, and reduced upon activation normal T cell expressed and secreted (RANTES) (5). IL-27-treated THP-1 cells in the presence of lipopolysaccharide (LPS) stimulation had higher TNFα, IL-1β, and TLR4 generation, suggesting that IL-27 blocked the induction of endotoxin tolerance and upregulated TNFα secretion in tolerized THP-1 cells (5, 6). THP-1 cells treated with IL-27 induced the phosphorylation of p38 and extracellular regulated protein kinases (ERKs) and greater NF-κB/activator protein-1 (AP-1) activity (4). The addition of TLR7 and/or TLR8 agonists led to greater phosphorylation of p38 and ERK, and more NF-κB/AP-1 activity. Pretreatment of THP-1 cells with IL-27 in the presence of TLR7 and/or TLR8 agonists led to more TNFα, IL-6, IL-8, and RANTES expression (4). Similarly, monocytes from healthy controls were stimulated with IL-27, showing the increased induction of surface bone marrow stromal cell antigen 2 (BST-2) and the elevated expression of IL-6, IP-10, macrophage inflammatory protein-1α (MIP-1α), MIP-1β, and TNFα (7). Monocytes treated with IL-27 activated STAT3 and STAT1. IL-27rα Fc chimera, also called WSX-1 Fc chimera, is a kind of neutralizing antibody for IL-27. The addition of IL-27rα Fc chimera inhibited the phosphorylation of STAT1 and STAT3 and downregulated the expression of IP-10 (7). Monocytes from patients with sickle cell anemia were stimulated with heme, showing the elevated expression of IL-8 (8). By contrast, the addition of IL-27 to monocytes from patients with sickle cell anemia inhibited the production of IL-8 (8). Stimulation of monocytes with IL-27 induced the expression of STAT1 target genes suppressor of cytokine signaling 1 (SOCS1), interferon regulatory factor 1 (IRF1), and IP-10. IL-10 induced STAT1 phosphorylation in monocytes, whereas the addition of IL-27 inhibited IL-10-mediated induction of SOCS3, prostaglandin dehydrogenase (PGDH), decidual protein-induced progesterone (DEPP), CD163, and complement receptor 1 (CR1) (9). Monocytes pretreated with the NF-κB inhibitor and followed up with IL-27 stimulation show reduced expression of IL-6, IP-10, MIP-1α, MIP-1β, and TNFα (7). Monocytes treated with IL-27 in the presence of LPS led to the increased expression of IL-1β, TNFα, MIP-1α, and MIP-1β (6, 10). The addition of caspase-1 inhibitor, glybenclamide/CRID3 sodium salt, or probenecid abrogated increased IL-1β expression, suggesting that IL-27-enhanced, LPS-mediated IL-1β production requires nod-like receptor pyrin domain containing 3 (NLRP3), P2X7 receptor, and caspase-1 (6). The administration of Janus kinase 2 (JAK2) inhibitor, STAT1 inhibitor, STAT3 inhibitor, or NF-κB inhibitor in IL-27+LPS-treated monocytes significantly inhibited pro-inflammatory cytokine production, indicating that IL-27 enhances LPS-induced pro-inflammatory cytokine generation via JAK2/STAT1/STAT3/NF-κB signaling (10). Human monocytes treated with neutralizing anti-IL-27 antibody (Ab) led to the elevated expression of granulocyte-macrophage colony-stimulating factor (GM-CSF), IL-1α, IL-1β, IL-6, MIP-1β, IL-23, TNFα, and IL-10, and increased the activation of inhibitor of the NF-κB (IκB)/NF-κB pathway (11). The addition of IκB kinase inhibitor blocked IL-1β and IL-6 expression. When CD4^+^ T cells were co-cultured with monocytes with neutralizing antibody to IL-27, there was elevated frequency of IL-17A-producing cells (11), suggesting that IL-27 acts as an endogenous constitutive repressor of monocytes. The anti-inflammatory role of IL-27 may correlate with the usage of polypropylene plates, which may reduce background activation (11). When IL-27-treated monocytes were cultured with autologous natural killer (NK) cells, IFNγ production was decreased, and NK cells preserved the ability to secrete lytic granules (12). IFNγ-treated human monocytes showed an increase in C-C motif chemokine ligand 2 (CCL2), CXCL9, IL-27, and IP-10 production (13). IFNγ-treated mice showed an increase in CXCL9 expression in intermediate monocytes and an increase in IL-27 expression in classical, intermediate, patrolling monocytes. After coculturing with IFNγ-treated monocytes in the presence of neutralizing antibody to IL-27, there was inhibited NK cell expansion (13). All these indicated that IL-27 may mainly play a pro-inflammatory role in monocytes and mediate an inhibitive role for NK cells (**Figure 1**).

### 2.2 IL-27 both inhibits and promotes the function of macrophage

**Figure 2:**
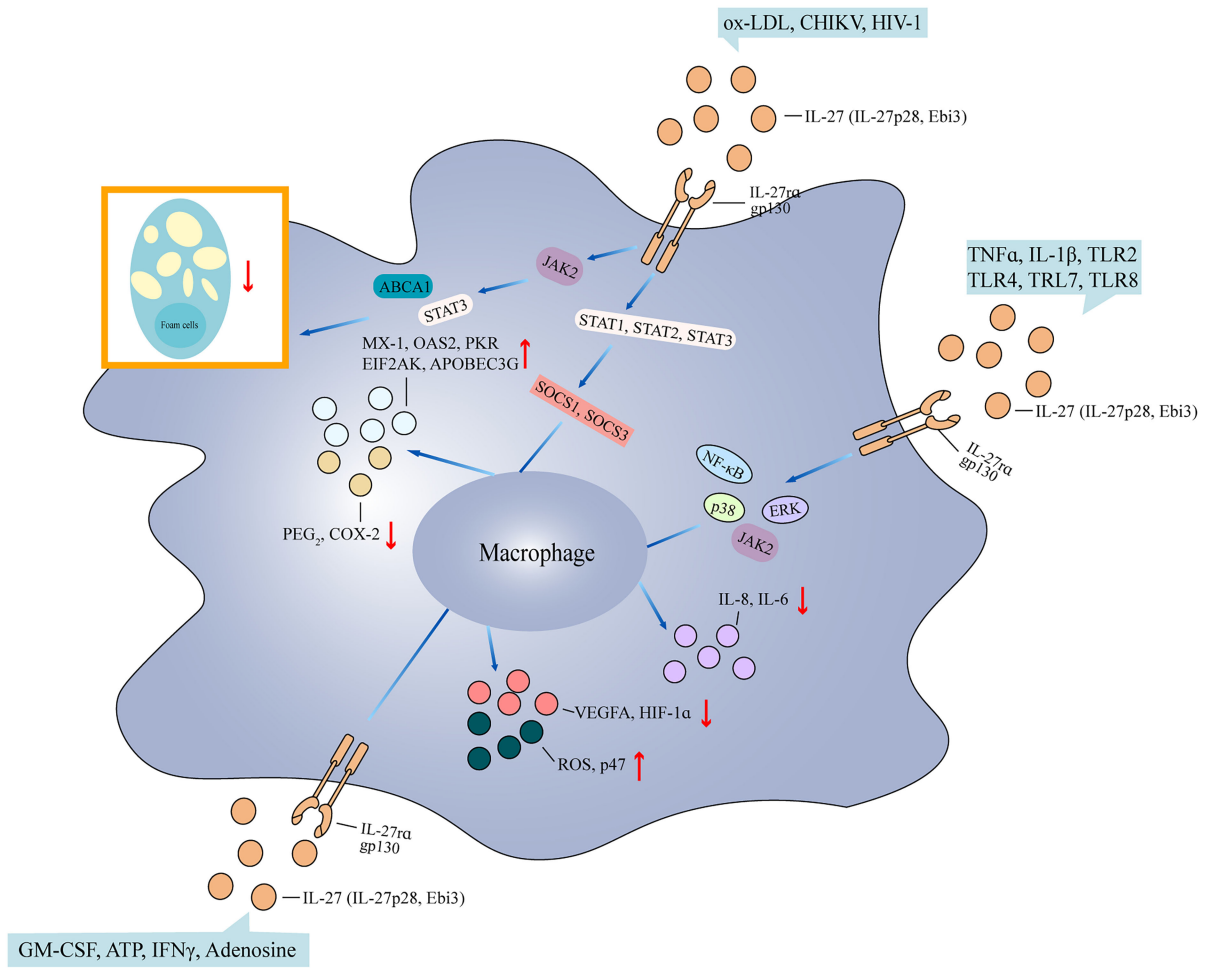
Impact of IL-27 on macrophage. Pathways regulated by IL-27 in the cytoplasm, and IL-27 linked to multiple biological effects in the nucleus. (1) After ox-LDL stimulation or CHIKV and HIV-1 infection, IL-27-treated macrophages activate JAK2 signaling, which then leads to ABCA1 and STAT3 activation, promoting foam cell formation. On the other hand, the signaling results in STAT1/STAT2/STAT3 activation, which synergizes with SOCS1/SOCS3 pathways, promote the transcription of MX-1, OAS2, PKR, EIF2AK, and APOBEC3G, or inhibit PGE_2_ and COX-2. (2) External stimulation including TNFα, IL-1β, TLR2, TRL4, TLR7, and TLR8 on IL-27-treated macrophages activates signaling NF-κB, ERK, p38, and JAK2, and then downregulates expression of IL-8 and IL-6. (3) GM-CSF, APT, IFNγ, and adenosine stimulate macrophages in the presence of IL-27, which leads to JAK2, PI3K, Akt, and mTOR activation, and finally inhibits VEGFA and HIF-1α expression but increases ROS and p47 generation. ABCA1, ATP binding cassette transporter A1; MX-1, myxovirus resistance protein 1; OAS2, 2’-5’-oligoadenylate synthetase 2; PKR, protein kinase R; EIF2AK, eukaryotic translation initiation factor 2 alpha kinase; APOBEC3G, apolipoprotein B mRNA editing enzyme catalytic subunit 3G; PGE_2_, prostaglandin E2; COX-2, cyclooxygenase 2; VEGFA, vascular endothelial growth factor A; HIF-1α, hypoxia-inducible factor-1 alpha; ROS, reactive oxygen species; PI3K, phosphoinositide 3-kinase; Akt, protein kinase B; mTOR, mammalian target of rapamycin; ox-LDL, oxidized-low density lipoprotein; CHIKV, chikungunya virus; GM-CSF, granulocyte-macrophage colony-stimulating factor.

Macrophages are another kind of innate immune cells that have different functions in innate immune responses, such as maintaining tissue homeostasis, antigen presentation, and killing antigens. U937 cells treated with PMA became M2 phenotype macrophages, showing upregulated adhesion and aggregation of U937 cells with mixed spindle and round cell morphologies, increased expression of CD68 and CD206, and reduced expression of CD86 and IL-12p40 (14). After stimulation with IL-27, the M2 macrophages had reduced expression of CD206 and increased expression of CD86 and IL-12p40, suggesting that IL-27 promoted M1 phenotype macrophage (14). THP-1-derived macrophages treated with oxidized low-density lipoprotein (ox-LDL) resulted in foam cell formation, evidenced by heavy lipid loading (15). The addition of IL-27 downregulated foam cell formation and the expression of ATP binding cassette transporter A1 (ABCA1) and increased cholesterol efflux and the phosphorylation expression of STAT3. However, the addition of neutralizing antibody to IL-27 upregulated foam cell formation and lipid accumulation. The ABCA1 promoter transfected with STAT3 led to repression of ABCA1. Stimulation with JAK2 inhibitor downregulated cellular cholesterol, free cholesterol, and cholesterol ester in IL-27-treated cells. Therefore, IL-27 suppresses foam cell formation by promoting macrophage ABCA1 expression through the JAK2/STAT3 pathway (15). THP-1-derived macrophages infected with chikungunya virus (CHIKV) induced pro-inflammatory and antiviral programs in macrophages, and activated SOCS1, SOCS3, STAT1, STAT2, and STAT3 signaling. The addition of IL-27 led to a decrease in CHIKV replication (16). IL-27rα-expressing RAW264.7 cells stimulated with IL-27 showed increased phosphorylation expression of STAT1 and STAT3, and produced low levels of prostaglandin E2 (PGE_2_) and cyclooxygenase 2 (COX-2). STAT1 knockdown in RAW264.7 cells promoted COX-2 expression (17). Moreover, monocyte-derived macrophages (MDMs) from healthy controls treated with IL-27 induced the expression of antiviral genes myxovirus resistance protein 1 (MX-1), 2’-5’-oligoadenylate synthetase 2 (OAS2), protein kinase R (PKR)/eukaryotic translation initiation factor 2 alpha kinase (EIF2AK), and apolipoprotein B mRNA editing enzyme catalytic subunit 3G (APOBEC3G), and activated STAT1 signaling (18). MDMs treated with TNFα induced IL-8 expression and activation of IκBα, ERK, and p38 (19). MDMs stimulated with IL-1β produced mature IL-1β protein and activated IκBα, ERK, and p38. When MDMs were exposed to IL-27, IL-8 expression was attenuated. The addition of IL-27 to TNFα-stimulated MDMs inhibited IκBα, ERK, and p38 phosphorylation, and downregulated the expression of the second receptor for TNFα, p75. Interestingly, the production of the cleaved form of IL-1β protein in response to IL-1β stimulation was abolished after MDMs were treated with IL-27, and there was reduced expression of IL-6 and IL-8 and less activation of IκBα, ERK, and p38 in MDMs treated with IL-1β+IL-27. MDMs exposed to IL-27 generated higher expression of IL-6, IL-1β, and TNFα in the presence of different TLR ligands’ stimulation, including TLR2, TLR4, and TLR7/8 ligands, suggesting that IL-27 inhibits TNFα and IL-1 signaling (19). MDMs had increased vascular endothelial growth factor A (VEGFA) production upon stimulation with M-CSF, adenosine, or ATP. The addition of IL-27 downregulated the production of VEGFA and hypoxia-inducible factor-1 alpha (HIF-1α), but produced more reactive oxygen species (ROS) and p47^phox^ (20, 21). p47^phox^ knockdown in MDMs had low expression of p47^phox^ and ROS, whereas overexpression of p47^phox^ in MDMs induced significant superoxide production, revealing that IL-27 enhances the potential of ROS production by induction of p47^phox^ (21). MDMs cultured with human AB serum in the presence of IL-27 induced autophagy (22). After HIV-1 virus infection, human AB serum+IL-27-treated MDMs restricted HIV-1 replication, and had accumulation of autophagosomes in MDMs, suggesting that IL-27-induced autophagy may restrict HIV-1 infection (22). MDMs infected with HIV-1 virus showed robust spreading infection, whereas there was little replication in MDMs treated with IL-27 after HIV-1 virus infection (23). MDMs infected with HIV-1 virus in the presence of IL-27 had a reduction in β2-spectrin (SPTBN1) expression. Silencing SPTBN1 in MDMs abrogated HIV-1 virus infection, indicating that IL-27 inhibits HIV-1 infection in macrophages by downregulating SPTBN1 (23). MDMs infected with *Mycobacterium tuberculosis* in the presence of IFNγ induced the generation of autophagosomes, phagosome maturation, and autophagy and phagosomal acidification, whereas the addition of IL-27 inhibited IFNγ-mediated effects (24). MDMs pretreated with JAK2 inhibitor and then treated with IFNγ and IL-27 showed no inhibition of puncta formation. IL-27 activated the protein kinase B (Akt)/mammalian target of rapamycin (mTOR) pathway and upregulated the phosphorylation expression of p70S6 K and 4E-BP. Therefore, IL-27 suppresses IFNγ-induced autophagy by induction of the JAK2/PI3K/Akt/mTOR cascade in *M. tuberculosis*-infected macrophages (24). When MDMs were infected with bacille Calmette-Guerin and then were treated with sIL-27R, there was reduced bacterial burden (25). Bone marrow cells isolated from IL-27rα^−/−^ and WT mice were differentiated into macrophages (BMDMs). After stimulating with LPS, there were low levels of IL-1β in BMDMs from both WT mice and IL-27rα^−/−^ mice and increased expression of PGE_2_ and COX-2 (6, 17). When BMDMs were exposed to LPS and ATP, IL-1β expression was decreased in IL-27rα^−/−^ mice (6). BMDMs from Ebi3^−/−^ mice and IL-27rα^−/−^ mice showed greater uptake of DiI, and higher expression of monocyte chemoattractant protein-1 (MCP-1), IFNγ, IL-1β, and IL-6 than those from controls. Treatment with IL-27 inhibited the uptake of DiI and the production of cytokines in BMDMs from LDL receptor^−/−^Ebi3^−/−^ mice (26). When BMDMs from IL-27rα^−/−^ mice were cocultured with naive CD4^+^ T cells, there was increased expression of IFNγ and IL-17A. However, the addition of EP2 and EP4 antagonists significantly inhibited the expression of IFNγ and IL-17A (17). Together, IL-27 regulates the pro-inflammatory cytokine production and antigen presentation ability of macrophages (**Figure 2**).

### 2.3 IL-27 has a dual role in dendritic cells

**Figure 3:**
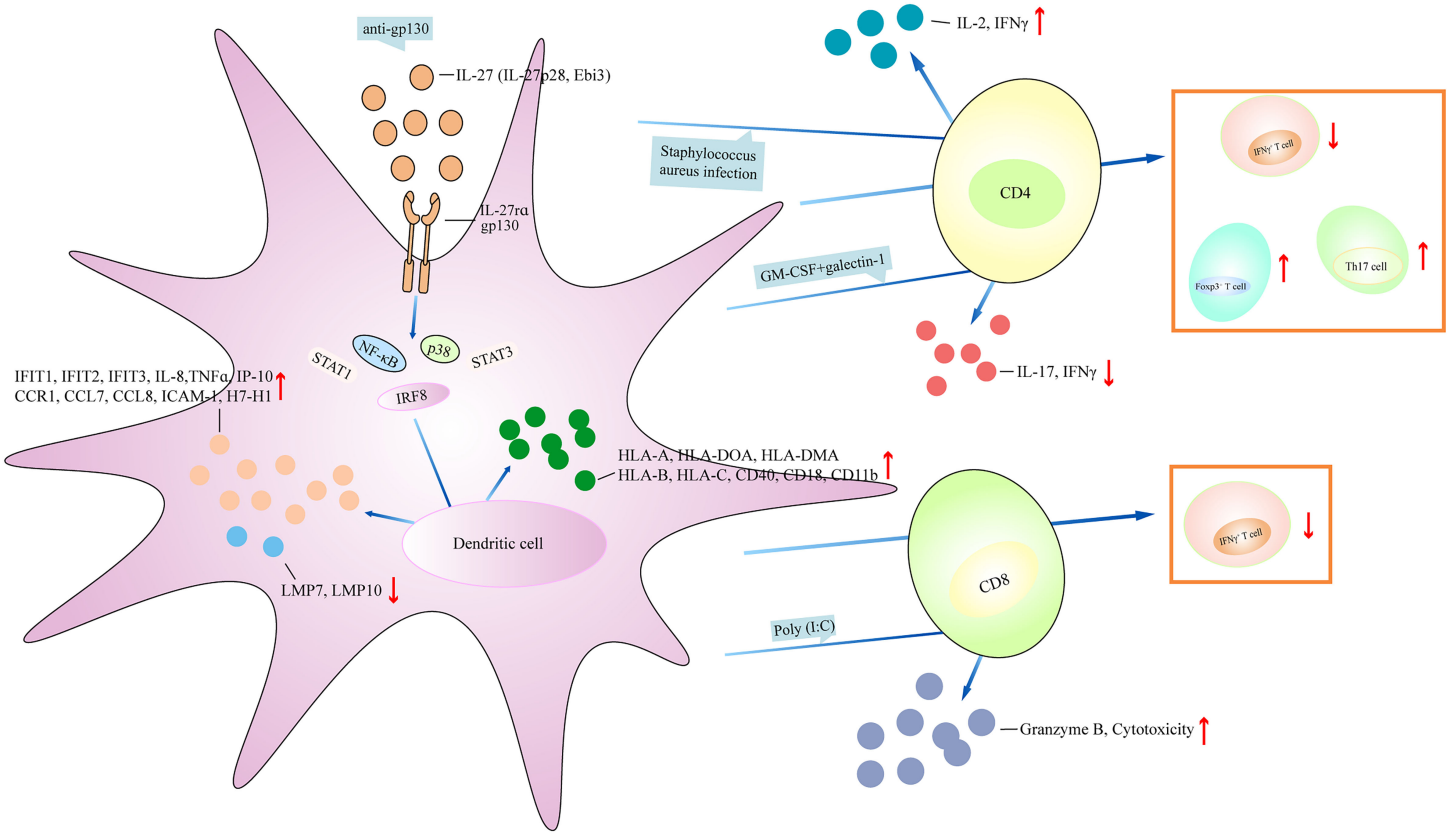
IL-27 regulates dendritic cell function. IL-27 affects the generation of inflammatory cytokines and chemokines, regulates the expression of surface markers on dendritic cell, and plays a central role in controlling multiple regulatory activities. (1) IL-27 activates NF-κB, STAT1, STAT3, p38, and IRF8 signaling directly or indirectly (mediated by anti-pg130), which then inhibit the production of IFIT1, IFIT3, IL-8, TNFα, IP-10, CCR1, CCL7, ICAM-1, HLA-A, HLA-DOA, CD40, and CD18, and upregulates the expression of MLP7 and LMP10. (2) IL-27 promotes IL-2 and IFNγ expression after *S. aureus-*infected dendritic cell coculturing with CD4^+^ T cell, and inhibits the expression of IL-17 and IFNγ under GM-CSF+galectin-1-stimulated dendritic cell coculturing with CD4^+^ T cell. IL-27 has been linked to increasing Foxp3^+^ T cell, Th17 cell, and reducing IFNγ^+^ T cell after coculturing dendritic cell and CD4^+^ T cell. Moreover, dendritic cell antagonizes IFNγ^+^ T-cell development when dendritic cell cocultures with CD8+ T cell in the presence of IL-27, and IL-27-treated dendritic cell coculturing with CD8^+^ T cell under poly(I:C) stimulation has higher expression of granzyme B and cytotoxicity of CD8^+^ T cell. IFIT1, interferon-induced protein with tetratricopeptide repeats-1; CCR1, C-C chemokine receptor 1; CCL7, C-C motif chemokine ligand 7; ICAM-1, intercellular adhesion molecule-1; LMP7, low-molecular-mass peptide-7.

DCs are a unique myeloid cell lineage and are capable of initiating and directing immune responses. Human cord blood DCs and monocyte-induced DCs (moDCs) treated with IL-27 resulted in increased phosphorylation expression of STAT1, and elevated expression of IRF8 (27). IL-27-treated cord blood DCs showed higher expression of HLA-A, HLA-DOA, HLA-DMA, interferon-induced protein with tetratricopeptide repeat-1 (IFIT1), IFIT2, IFIT3, CD40, IL-8, TNFα, IP-10, C-C chemokine receptor 1 (CCR1), CCL7, and CCL8 than IL-27-treated moDCs (27). MoDCs cultured with IL-27 increased the migration of the cells; reduced the expression of tapasin, low-molecular-mass peptide-7 (LMP7), and LMP10; and increased HLA class I molecules (HLA-A, -B, and -C) (28). The addition of anti-gp130 neutralizing antibody significantly upregulated the expression of tapasin, LMP7, and LMP10, and downregulated the expression of HLA-A, -B, and -C. IL-27-treated moDCs cocultured with autologous CD8^+^ T cells or CD4^+^ T cells inhibited the percentage of IFNγ^+^ T cells (28). Human naive CD45RA^+^CD45RO^−^CD8^+^ T cells stimulated by IL-27 induced the proliferation of CD8^+^ T cells and cytotoxic activity, and enhanced the expression of T-bet and IFNγ, and poly (I:C)-primed DCs produced high levels of IL-27 (29). However, when naive CD8^+^ T cells were cocultured with poly (I:C)-primed DCs in the presence of neutralizing antibody against IL-27R, there was low expression of cytogranzyme B, which inhibited cytotoxicity, indicating that IL-27 regulates poly (I:C)-primed DCs to promote the function of CD8^+^ T cells (29). MoDCs treated with latex beads and lysotracker in the presence of IL-27 promoted the acidification of the latex beads (30). MoDCs treated with IL-27 upregulated the expression of HLA-DR, MHC class II, CD40, intercellular adhesion molecule-1 (ICAM-1), CD18, and CD11b. MoDCs treated with IL-27 and bafilomycin and then infected with *Staphylococcus aureus* showed cleared bacteria and increased the expression of IL-12. Coculturing IL-27-treated moDCs with allogeneic CD4^+^ T cells in the presence of *S. aureus* infection increased T-cell proliferation and IL-2 and IFNγ production (30). Thus, IL-27 promotes improved antigen processing and DC-mediated stimulation of T cells. In apoptotic tumor cell condition medium (ACM)-treated moDCs, IL-27 expression was upregulated (31). When ACM-treated moDCs were cultured with IL-27-neutralizing antibody, and then were cocultured with autologous T cells, there was increased cytotoxicity and reduced CD69 expression in CD39^+^ cells (31). By contrast, IL-27-treated moDCs showed upregulation of B7-H1 and increased phosphorylation expression of STAT1, whereas the effects were blocked by adding anti-IL-27 neutralizing antibody (32). IL-27-treated moDCs had reduced potential to activate allogeneic CD4^+^ T cells, showing low expression of IFNγ and IL-2 (32). IL-27rα^−/−^ mice infected with *Trypanosoma congolense* or *Trypanosoma brucei* showed higher frequency of moDCs and TNF/inducible nitric oxide synthase (iNOS)-producing DCs (Tip-DCs) in the liver (33). When transferring Ly6C^−^ monocytes into infected IL-27rα^−/−^ mice, there was no significant accumulation of Ly6C^+^ monocytes in the liver, and reduced frequency of moDCs and Tip-DCs. Interestingly, when Ly6C^+^ monocytes were differentiated into DCs, the addition of Ly6C^−^ monocytes inhibited Ly6C^+^ monocyte differentiation into Tip-DCs, suggesting that the development of Tip-DCs in infected IL-27rα^−/−^ mice was suppressed by Ly6C^−^ monocytes (33). Liver and spleen plasmacytoid DCs (pDCs) treated with IL-27 revealed elevated expression of B7-H1 and reduced expression of CD86 (34). The addition of STAT3 inhibitor decreased B7-H1 expression. Coculturing IL-27-treated pDCs with allogeneic splenic CD4^+^ T cells promoted the percentage of Foxp3^+^ cells. When B7-H1^−/−^ liver pDCs were cocultured with allogeneic splenic CD4^+^ T cells in the presence of IL-27, the percentage of CD4^+^Foxp3^+^ T cells was decreased. Liver and spleen pDCs from Ebi3^−/−^ mice stimulated with CpG type B showed low expression of B7-H1, IL-27rα, and CD86. Ebi3^−/−^ pDCs cultured with CD4^+^ T cells induced CD4^+^ T-cell proliferation and upregulated the expression of IFNγ (34). WT mice bone marrow cells stimulated with GM-CSF+galectin-1 resulted in the low expression of CD11c and the high expression of CD45RB, IL-27p28, Ebi3, IL-6, and IL-10 (35). Coculturing alloreactive CD4^+^ T cells with GM-CSF+galectin-1-induced bone marrow monocyte-induced DCs (BMDCs) led to less proliferation of T cells and reduced the expression of IFNγ and IL-17. However, inhibiting IL-27 expression abrogated the effects, suggesting that IL-27 regulates the galectin-1-mediated tolerogenic function of DCs (35). Mice with IL-27rα^−/−^ DCs showed low expression of CD80 and CD86 (36). After LPS stimulation, the expression of CD80 and CD86 and the phosphorylation expression of STAT3, p38, and IκB in the DCs increased, and the percentage of CD80^+^ cells and the expression of TNFα, IL-12p70, IL-12p40, Ebi3, Delta-4, and Jagged-1 were upregulated in the DCs. Coculturing IL-27rα^−/−^ DCs with WT CD4^+^ T cells in the presence of LPS promoted T-cell proliferation and upregulated the production of IFNγ. Similarly, coculturing IL-27rα^−/−^ DCs with WT NK cells in the presence of LPS promoted killing activity and upregulated the production of IFNγ and TNFα. Therefore, IL-27 deficiency in DCs augments the antigen-presenting and Th1-promoting function (36). IFNγ^−/−^ mice showed lower expression of IL-27 as compared to that in WT mice (37). T cells from IFNγ^−/−^ mice showed higher expression of IL-9 compared with that in WT T cells. Injection of neutralizing IL-27 antibody upregulated IL-9 production in IFNγ^−/−^ mice, and adding conditioned medium (CM) from IFNγ-treated DCs into Th9 cells inhibited IL-9 expression. In contrast, adding CM from IFNγ+anti-IL-27 antibody-treated DCs into Th9 cells upregulated IL-9 production, indicating that IL-27 in DCs regulates IFNγ-mediated limitation of T helper 9 (Th9) cell differentiation (37). Moreover, when naive CD4^+^ T cells from WT rats were co-cultured with WT DCs, T cells were much more proliferated, and Th17 cells were differentiated (38). The addition of galectin-1 weakened the effects of DCs, evidenced by reduced expression of IL-17 and less Th17 cells. However, further adding anti-IL-27 antibody promoted T-cell proliferation and upregulated IL-17 expression, suggesting that galectin-1 regulates DC-induced Th17 balance via IL-27 (38). Together, IL-27 significantly regulates inflammatory cytokine production in DCs and the antigen-presenting ability of DCs. Based on the above findings, the role of IL-27 in DCs was partly different. Some studies suggest an inhibitory role of IL-27 in DCs (32, 36), whereas other studies indicate a positive role of IL-27 in DCs (27, 28, 34, 38). This may correlate with several reasons. First, DCs were generated from different species, including humans, mouse models, and rats. Second, a different microenvironment where IL-27 regulates DC function may affect the role of IL-27 in DCs. Third, IL-27 interacts with different downstream signaling to form a different regulatory network, which will differently regulate DCs’ function (**Figure 3**).

### 2.4 IL-27 plays an anti-inflammatory role in neutrophil

Neutrophils are surveillance cells, and are the first responders against infectious or inflammatory challenges. Neutrophils isolated from healthy controls were stimulated with fMLP in the presence of IL-27, showing suppressed adhesion activity and decreased expression of macrophage-1 antigen (Mac-1) (39). LPS-treated neutrophils showed high expression of ROS and neutrophil enzyme peroxidase (POX), whereas adding IL-27 significantly inhibited LPS-induced ROS and POX production in human neutrophils and upregulated the expression of IL-1β (39). Neutrophils infected with *Burkholderia pseudomallei* showed high expression of IL-27p28 and IL-27rα (40). The addition of IL-27 upregulated the number of intracellular bacteria surviving and reduced oxidative burst and ROS production. Neutrophils pretreated with JAK2 inhibitor and then infected with *B. pseudomallei* led to the reduction of oxidative burst and upregulated the survival of intracellular bacteria. When neutrophils were infected with *B. pseudomallei* in the presence of sIL-27rα, there was a reduced number of bacteria surviving and low expression of IL-1β (40). Neutrophils isolated from hemoglobin SS (HbSS) patients were stimulated with heme in the presence of IL-27, showing the low expression of IL-8 (8). Polymorphonuclear neutrophils (PMNs) isolated from intracerebral hemorrhage (ICH) rats stimulated with IL-27 revealed reduced inducible nitric oxide synthase (iNOS), matrix metallopeptidase-9 (MMP-9), and NADPH oxidase 2 (NOX2); increased hemoglobin-neutralizing Hp and iron-sequestering LTF, IL-27rα, and gp130; and increased the phosphorylation expression of STAT3 (41). When IHC rats were injected with anti-IL-27 antibody, there was increased expression of pro-inflammatory factors and reduced expression of Hp, LTF, and IL-27R (41). WT mice injected with zymosan resulted in increased recruitment of GR1^+^/CD11b^+^ neutrophils in the peritoneal cavity (42). The addition of IL-27 reduced neutrophil numbers and reduced the expression of IL-17, CXCL1, CCL2, and CCL3 in the peritoneal cavity. Peripheral blood neutrophils stimulated with zymosan reported an increase in neutrophil numbers, whereas further treatment with IL-27 led to a reduction of neutrophils in the blood (42). Ebi3^−/−^ mice infected with *Leishmania infantum* showed high expression of IL-17 and CXCL1 and increased neutrophil migration in both the spleen and the liver (43). Infection of IL-17rα^−/−^ mice with *L. infantum* revealed less frequency of neutrophils in the spleen. Ebi3^−/−^ mice injected with anti-IL-17A antibody abrogated host resistance and mitigated neutrophil influx (43). Furthermore, the number of CD11b^+^Gr-1^+^ neutrophils in the lungs of IL-27rα^−/−^ mice was higher than that in the lungs of WT mice, and IL-27rα^−/−^ mice had increased expression of MMP-8 and S100A8, two neutrophil-derived proteins related to lung inflammatory response (44). IL-27rα^−/−^ mice administered anti-IL-17 antibody revealed a lower number of neutrophils and reduced expression of MMP-8 and S100A8 (44). Collectively, IL-27 was a negative regulator of neutrophil function.

### 2.5 IL-27 promotes natural killer cell effector function

NK cells are a population of innate lymphocytes, which have an intrinsic ability to identify and eliminate cancer and virally infected cells. NK cells constitute approximately 10%–15% of lymphocytes in the immune system and are mainly involved in the clearance of tumor cells and virus-infected cells. Cord blood NK cells (CD16^+^CD56^-^ and CD16^+^CD56^+^) stimulated with IL-27-promoted NK cells aggregate and induced the activation of STAT3 (45). CD3^-^CD56^+^ NK cells from PBMCs of healthy controls were cultured with IL-27, revealing more NK cell expansion; increased the expression of CD107a, TNF-related apoptosis inducing ligand (TRAIL), natural killer group 2D (NKG2D), NK cell p44-related protein (NKp44), NKp30, CD226, CD158b, CD96, and CD69; and reduced the expression of NKp46 (46, 47). IL-27 stimulation increased the size and cytoplasmic granularity of NK cells, and upregulated the expression of granzyme B and perforin in NK cells (46). Human NK cells stimulated with IL-27 produced increased expression of IFNγ (47). The addition of mitogen-activated protein kinase kinase 1 (MEK1), MEK2, c-Jun N-terminal kinase (JNK), phosphoinositide 3-Kinase (PI3K), mTOR, NF-κB, and STAT1 inhibitors significantly inhibited IFNγ expression. IL-27 stimulation promoted NK cell activation, evidenced by elevated expression of CD25, and upregulated the percentage of degranulating NK cells. Stimulation of NK cells with IL-18 induced high expression of IFNγ, and the addition of IL-27 induced higher expression of IFNγ and T-bet in NK cells. NK cells stimulated with both IL-27 and IL-18 exhibited increased cytotoxicity, whereas inhibiting NKp46 and TRAIL expression suppressed cytotoxicity (47). When cord blood NK cells were co-cultured with BJAB lymphoma target cells in the presence of IL-27, the cytotoxic activities of NK cells were enhanced (45). CD3^-^CD56^+^ NK cells from peripheral blood mononuclear cells (PBMCs) of healthy controls were cocultured with K562, A2780, Raji, T-47D, and HCT116 cells in the presence of IL-27, and the cytotoxic activities of NK cells were elevated as well (46, 47). This was similar to NK cells coculturing with the EA cell line, by which adding IL-27 to the culture system significantly upregulated the ability of NK cell-mediated killing of EA cells and increased the expression of CD69, NKG2D, and NKp46 receptor (48). Stimulation of CD56^bright^ and CD56^dim^ NK cells with IL-27 upregulated the expression of IL-10 and IFNγ and the viability of NK cells, and decreased the proliferation of the cells (49). Coculturing IL-27-treated CD56^bright^ or CD56^dim^ NK cells with autologous CD4^+^ T cells showed that IL-27-treated CD56^bright^ NK cells significantly suppressed CD4^+^ T-cell proliferation and increased the expression of perforin as compared to those in CD56^dim^ NK cells (49). IL-27p28 interacted with cytokine-like factor 1 (CLF) to form the p28/CLF complex, which stimulated CD3^-^CD56^+^ NK cells to produce more IFNγ, CD54, and CD69 (50). Transfecting the pro-B cell line Ba/F3 with gp130 in the presence of p28/CLF induced STAT3 phosphorylation (50). Moreover, bone marrow-derived NK (BM-NK) cells from WT mice stimulated with IL-27 had increased expression of CD69 and percentage of degranulation on BM-NK cells (51). IL-27-treated BM-NK cells cocultured with K562 cells revealed an elevation of BM-NK cell cytotoxicity (51). Splenic CD4^−^CD8^−^B220^+^DX-5^+^ cells isolated from WT mice under IL-27 stimulation induced the phosphorylation of STAT1 and STAT3, and upregulated the expression of T-bet and granzyme B (52). The addition of sIL-27rα blocked IL-27-mediated effects. Splenic DX-5^+^ cells isolated from tumor-bearing mice were treated with IL-27, showing increased cytotoxicity against NK-sensitive YAC-1 cells. When the mice bearing an SCCVII tumor were injected with IL-27, splenic DX-5^+^ NK cells killed more target cells (52). Eca109 cells expressing IL-27 were injected into WT mice, displaying increased expression of IFNγ, T-bet, IL-12Rβ2, TNFα, IL-1β, and IL-6 and upregulated activities to target YAC-1 cells (53). WT mice infected with the A/PR/8/34 H1N1 (PR8) influenza virus showed increased percentage of IFNγ^+^ NK cells and expression of IL-27rα in NK cells (54). IL-27rα^−/−^ mice infected with influenza showed a reduction in the percentage of CD27^+^CD11b^+^ NK cells in the alveolar space and in the bone marrow and less NK T cells were recruited to the bronchoalveolar space. There was elevated expression of Ahr (Aryl hydrocarbon receptor), cyclin D1, Elk1 (ETS domain-containing protein), and CD117, and reduced expression of forkhead box 1 (Foxo1), Foxo4, IRF4, Myc, nuclear factor of activated T cell (NF-AT), T-bet, nuclear respiratory factor 1 (Nrf1), Nrf2, mitochondrial transcription factor A (TFAM), carnitine palmitoyltransferase 1 (CPT1), and v-Maf musculoaponeurotic fibrosarcoma oncogene (Maf) homolog F (MafF) in IL-27rα^−/−^ mice infected with influenza. IL-27-stimuated NK cells showed activation of NKG2D. Transferring splenocytes from IL-27rα^−/−^ mice into lymphocyte-deficient Rag2^−/−^γc^−/−^ mice followed by influenza infection showed lower expression of IFNγ (54). Collectively, IL-27 promotes NK cell effector functions.

### 2.6 IL-27 regulates the function of eosinophil and inhibits the function of mast cell

Eosinophils are a subset of bone marrow-generated granulocytes, which are mainly involved in allergic diseases. Mast cells are tissue-resident hematopoietic cells and work in numerous immune responses, such as allergic reaction, innate immunity, and autoimmunity. Expression of gp130 was highly expressed on eosinophils from healthy controls and eosinophils stimulated with IL-27 induced STAT1, ERK, JNK, p38, and IκBα phosphorylation; upregulated the percentage of viable eosinophils; and downregulated Annexin V^+^ population (55). Similarly, incubation of eosinophils with IL-27 increased the surface expression of CD18 and ICAM-1, and decreased the expression of L-selectin. IL-27 stimulation increased the number of eosinophils adhered to fibronectin-coated wells. IL-27-treated eosinophils processed elongated shape and aggregated together, and showed a significant increase in band shift. Upon IL-27 stimulation, eosinophils showed high expression of IL-6, TNFα, IL-1β, CCL2, CXCL8, and CXCL1. WT mice were sensitized and challenged with ovalbumin (OVA), and then were injected with IL-27, showing a lower number of eosinophils in the nasal mucosa (56). IL-27rα^−/−^ mice infected with Sendai virus experienced increased weight loss, more severe lung lesions, and elevated number of pulmonary eosinophils (57). All the findings revealed that IL-27 may regulate eosinophils by stimulating downstream signaling, such as NF-κB and MAPK. For the study discussed mast cells, IL-27rα^−/−^ mice sensitized with anti-DNP IgE antibody+DNP-BSA showed much more body temperature drops, elevated hypersensitivity reaction, and more severe ear swelling (58). When IL-27rα^−/−^ mice were sensitized with OVA and then followed by intranasal OVA challenges, there was a higher infiltration of inflammatory cells (including mast cells) and hyperplasia of mucus-secreting goblet cells (PAS^+^) in large bronchioles. Transferring WT mice bone marrow-derived mast cells (BMMCs) to IL-27rα^−/−^ mice inhibited the airway inflammation, and produced less inflammatory cytokines such as IL-4, IL-5, IL-13, IL-6, and TNFα. BMMCs from IL-27rα^−/−^ mice were stimulated with IL-27 and then sensitized with anti-DNP IgE and DNP-BSA, revealing enhanced activation of Lyn, phospholipase C gamma 1 (PLCγ1), Akt, IκBα, p38, and calcium flux signal, and less phosphorylation expression of src homology 2 domain-containing protein tyrosine phosphatase 1 (SHP1) and STAT3. There was reduced expression of histamine and CD107 from IgE-sensitized WT BMMCs in the presence of IL-27. Thus, IL-27 may inhibit mast cell activation and allergic responses.

## 3 Effect of IL-27 on adaptive immunity

### 3.1 IL-27 has multiple functions in T cells either in humans or in mouse models

#### 3.1.1 IL-27 promotes Th1 cell differentiation and function, but inhibits Th2 and Th17 cell function in humans

BST-2 is a membrane protein highly expressed in some tumors. It may be a potential target for cancer treatment. It was also considered as a host restriction factor that inhibited the release of enveloped viruses from host cells. In healthy controls, CD3^+^ T cells stimulated with IL-27 showed elevated expression of BST-2 (59). Naive and memory CD4^+^ T cells from healthy controls stimulated with IL-27 showed increased proliferation of naive and memory CD4^+^ T cells; upregulated cell division; G0/G1→S transition; activated SHP2, STAT1, STAT3, and STAT5 signaling; and promoted the expression of MHC class I, Pim-1, c-Myc, cyclin D2, cyclin D3, and CDK4 (60). The addition of c-Myc inhibitor or Pim-1 inhibitor suppressed IL-27-mediated proliferation and downregulated the expression of cyclin D2, cyclin D3, and CDK4 (60). Naive CD4^+^ T cells under Th17-polarizing conditions stimulated with IL-27 showed inhibited expression of IL-17, retinoic acid-related orphan receptor gamma t (RORγt), IL-22, IL-23R, CCR6, and CCL20 (61). Total CD4^+^ T cells from healthy controls stimulated with IL-27 displayed high expression of IFNγ and reduced expression of GATA-binding protein 3 (GATA-3) and RORγt (61). When total CD4^+^ T cells were stimulated with CD3/CD28, there was high expression of IL-17, IL-22, IL-23R, CCR6, and CCL20. However, the addition of IL-27 suppressed CD3/CD28-induced IL-17, IL-22, IL-23R, CCR6, and CCL20 expression (61). Naive CD4^+^ T cells stimulated with IL-27 induced IFNγ expression; activated STAT1, STAT3, and SOCS1; and downregulated the expression of IL-22 and the percentage of CD4^+^IL-22^+^ T cells; however, the addition of JAK2/STAT inhibitors suppressed IFNγ expression and inhibited SOCS1 activation (62, 63). Naive CD4^+^CD45RA^+^ T cells from healthy controls stimulated with IL-1β+IL-23 or IL-1β+IL-6+IL-23+TGF-β induced IL-17 expression, whereas the addition of IL-27 diminished IL-17 expression and the percentage of IL-17A^+^ cells and upregulated IFNγ secretion and the percentage of IFNγ^+^ cells (64). Naive CD4^+^ T cells stimulated with IL-27 under Th17-polarizing conditions showed low IL-22 expression and percentage of IL-22^+^ cells and upregulated the expression of SOCS1 and SOCS3. Naive CD4^+^CD45RA^+^ T cells stimulated with IL-27 activated STAT1 and STAT3, and upregulated the frequency of STAT1-expressing cells. Memory CD4^+^ T cells stimulated with IL-27 led to the low expression of IL-17 and RORγt and the low percentage of IL-17A^+^ cells (64). Similarly, naive and memory CD8^+^ T cells from healthy controls stimulated with IL-27 showed an increase of STAT1 and STAT3 phosphorylation (65). Total and naive CD8^+^ T cells stimulated with IL-27 induced SOCS1 and SOCS3 expression. Naive CD8^+^ T cells treated with IL-27 upregulated T-cell proliferation and the expression of T-bet, IFNγ, and granzyme B. Coculturing naive CD8^+^ T cells and mast cells in the presence of IL-27 showed a greater ability of CD8^+^ T cells to kill mast cells (65). Naive CD45RA^+^CD8^+^CCR7^+^ T cells stimulated with IL-27 induced the proliferation of the cells and the expression of IL-21, IFNγ, T-bet, and granzyme B, and upregulated the percentage of T-bet^+^, IL-21^+^, and T-bet^+^IL-21^+^ cells (66). Activated Vγ9Vδ2^+^ T cells cocultured with HTLA-230 cells and DAUDI cells in the presence of IL-27 showed more lysis of HTLA-230 cells and DAUDI cells, respectively (67). The addition of anti-TCR Vγ9 inhibited target cells’ lysis. Resting Vγ9Vδ2^+^ T cells stimulated with IL-27 upregulated the expression of granzyme A, IFNγ, and CD62L, and activated Vγ9Vδ2^+^ T cells stimulated with IL-27 promoted the expression of IL-5 and IL-13 (67). Moreover, naive CD4^+^ T cells were isolated from patients with autosomal dominant hyper IgE syndrome who had STAT3 gene mutation (68). After stimulating with IL-27, there was no induction of IL-21 expression (68). People living with HIV (PLWH) showed high expression of gp130 in total CD4^+^ T cells (69). IL-27rα monoclonal antibody (mAb) blocked STAT1 activation induced by IL-27. Anti-gp130 mAb blocked STAT3 activation induced by IL-6. Stimulation of naive, memory CD4^+^ T cells and naive CD8^+^ T cells from PLWH with IL-27 led to an increase of STAT1 phosphorylation. Total CD4^+^ and CD8^+^ T cells stimulated with IL-27 upregulated the expression of TBX21 and CD69 and downregulated the expression of GATA-3 and RORγt (69). T follicular helper (Tfh) cells sorted from patients with chronic hepatitis B (CHB) were primed with HBsAg, and then cocultured with autologous memory and naive B cells in the presence of IL-27 neutralizing antibody, showing more plasmablasts and plasma cell differentiation and less production of B lymphocyte-induced maturation protein-1 (Blimp-1) (70). Therefore, IL-27 promotes CD4^+^ T-cell differentiation into Th1 cells, inhibits Th2 and Th17 cell differentiation, and increases the cytotoxic activity of CD8^+^ T cells in humans.

#### 3.1.2 IL-27 promotes the differentiation and function of Th1 and Th2 cells and inhibits Th17, Th9, and Tfh cells in mouse models

Naive CD4^+^ T cells from WT mice treated with IL-27 showed decreased expression of IFNγ, IL-5, IL-17, GM-CSF, IL-1β, IL-3, MIP-1α, and MIP-1β; increased expression of IL-10, c-Maf, IL-21, ICOS, nuclear factor, interleukin 3 regulated (NFIL3), Tim-3, early growth response gene 2 (Egr-2), and gallectin-3; and higher phosphorylation expression of STAT1 and STAT3 (71–75). CD4^+^ T cells from STAT1^−/−^ and STAT3^−/−^ mice stimulated with IL-27 produced low expression of IL-10 (71, 76). CD4^+^ T cells from IL-27-injected WT mice showed an upregulated expression of T-bet, Eomes, Blimp-1, and kruppel-like factor 4 (KLF4) and a reduced expression of beta-catenin (CTNNB1) (77). CD4^+^CD25^−^ T cells stimulated with IL-27 promoted IL-10 expression, whereas the addition of PI3K inhibitor inhibited IL-10 expression (78). Egr-2^−/−^CD4^+^ T cells and Blimp-1^−/−^CD4^+^ T cells stimulated with IL-27 did not induce IL-10 expression but upregulated the expression of IFNγ and IL-17 (73). Egr-2 transfected to the Prdm1 transcription start site, leading to enhanced Prdm1 promoter activity. STAT3^−/−^CD4^+^ T cells stimulated with IL-27 did not induce Egr-2 expression, indicating that IL-27-induced Egr-2 expression in CD4^+^ T cells is dependent on STAT3 (73). Naive CD4^+^ T cells from WT mice treated with JAK2 and Tyk2 inhibitors abrogated IL-27-mediated inhibition of GM-CSF (74). Naive CD4^+^ T cells stimulated with IL-27 upregulated the recovery of viable T cells, and the percentage of cells that underwent activation-induced cell death (AICD), the percentage of activated caspase-8^+^ cells, and the expression of activated form of caspase-3 were reduced (79). The addition of anti-FasL mAb inhibited IL-27-mediated recovery of viable T cells. cFLIP is a homolog of caspase-8 that suppresses caspase-8 activation. When FAS-mediated AICD occurred, the addition of IL-27 restored the expression of cFLIP. IL-27 stimulation did not restore cFLIP expression in STAT3^−/−^CD4^+^CD45RB^high^ cells (79). Naive CD4^+^CD62L^high^CD25^−^ cells cultured with IL-27 induced IL-10, c-Maf, IL-21, IL-21R, and ICOS expression (75, 76). Adding a neutralizing IL-21 Ab downregulated the frequency of IL-10-producing T cells and IL-10 expression, and IL-21R^−/−^CD4^+^ T cells stimulated with IL-27 expressed low expression of c-Maf and IL-21 (75). WT and STAT3^−/−^ naive CD4^+^ T cells stimulated with IL-27 induced phosphorylation of STAT1 and STAT2, whereas IL-27 promoted T-cell proliferation, c-Myc, and Pim-1 expression in WT CD4^+^ T cells and did not promote the proliferation of STAT3^−/−^CD4^+^ T cells (80). Therefore, IL-27 downregulates pro-inflammatory cytokine production in WT CD4^+^ T cells by interacting with IL-21R, STAT1, STAT3, Blimp-1, and Egr-2.

**Figure 4:**
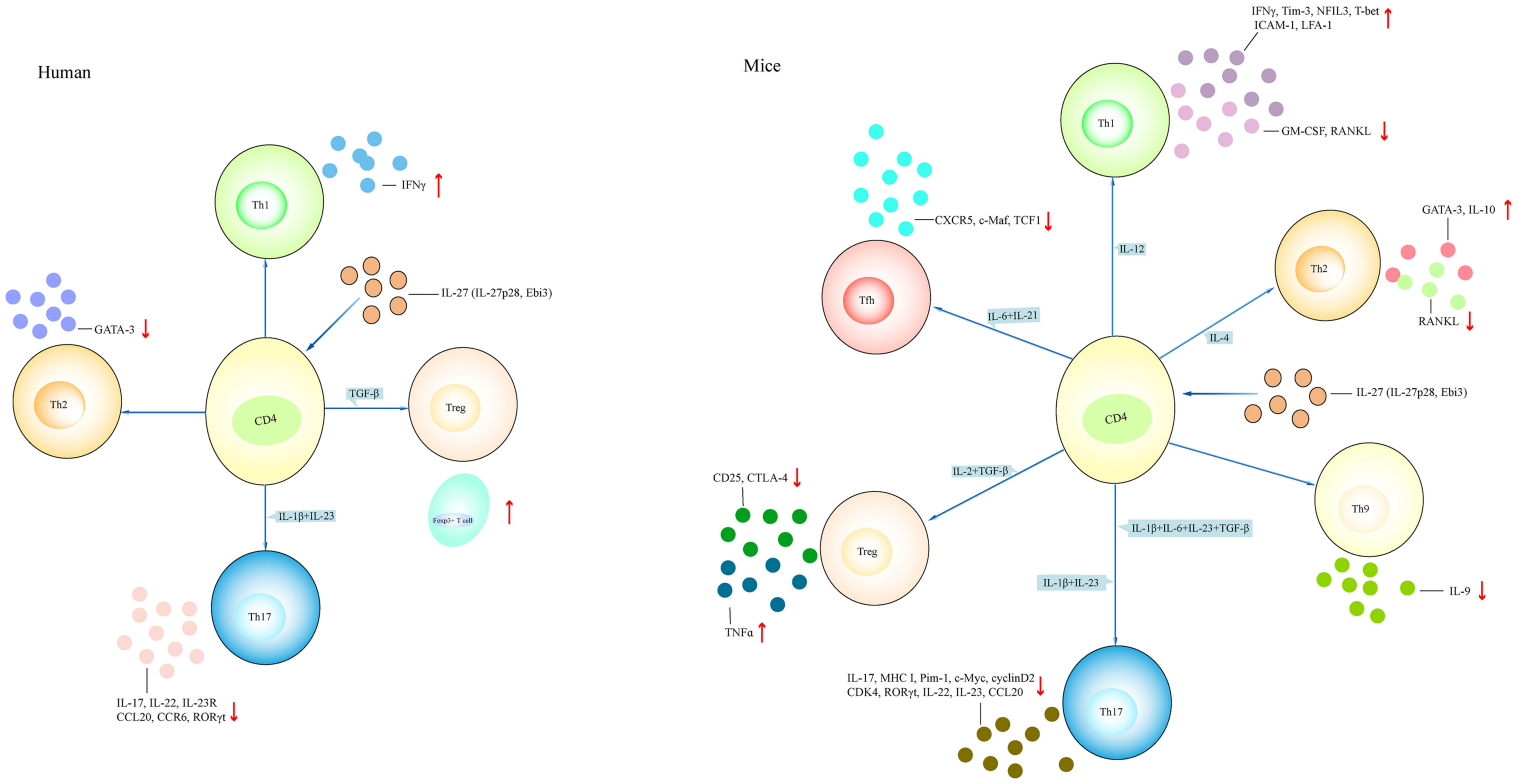
Different effects of IL-27 on T cell. The effect of IL-27 on CD4^+^ T cell in both mice and humans. IL-27 induces the differentiation of Th1 and Th2 cells and inhibits the differentiation of Th17, Treg, Th9, and Tfh cells in mouse models. IL-27 induces Th1 and Treg cell differentiation, and inhibits Th2 and Th17 cell differentiation in humans. (1) Naive CD4^+^ T cell treated with IL-27 in the presence of IL-12 promotes Th1-related cytokines such as IFNγ and NFIL3, and inhibits the expression of GM-CSF, RANKL. Naive CD4^+^ T cell treated with IL-4 in the presence of IL-27 increases the expression of GATA-3 and IL-10 and reduces the expression of RANKL. Naive CD4^+^ T cell treated with IL-1β+IL-6+IL-23+TGF-β or IL-1β+IL-23 in the presence of IL-27 downregulates the expression of IL-17, IL-22, and RORγt. Naive CD4^+^ T cell treated with IL-27 under Treg-polarizing conditions (IL-2+TGF-β) or Tfh-polarizing conditions (IL-6+IL-21) inhibits CD25 and CTLA-4 expression, or reduces CXCR5 and TCF-1 expression, respectively. (2) IL-27-treated CD4^+^ T cell under Th1-polarizing conditions or Treg-polarizing conditions has elevated IFNγ and Foxp3^+^ cell, respectively. IL-27-treated CD4^+^ T cell under Th2-polarizing conditions or Th17-polarizing conditions has reduced GATA-3 or IL-17, IL-22, and IL-23R, respectively. NFIL3, nuclear factor, interleukin 3 regulated; RANKL, receptor activator of nuclear factor-kappaB ligand; GATA-3, GATA-binding protein 3; CTLA-4, anti-cytotoxic T-lymphocyte antigen-4; CXCR5, CXC chemokine receptor type 5; TCF1, T cell factor 1.

**Figure 5:**
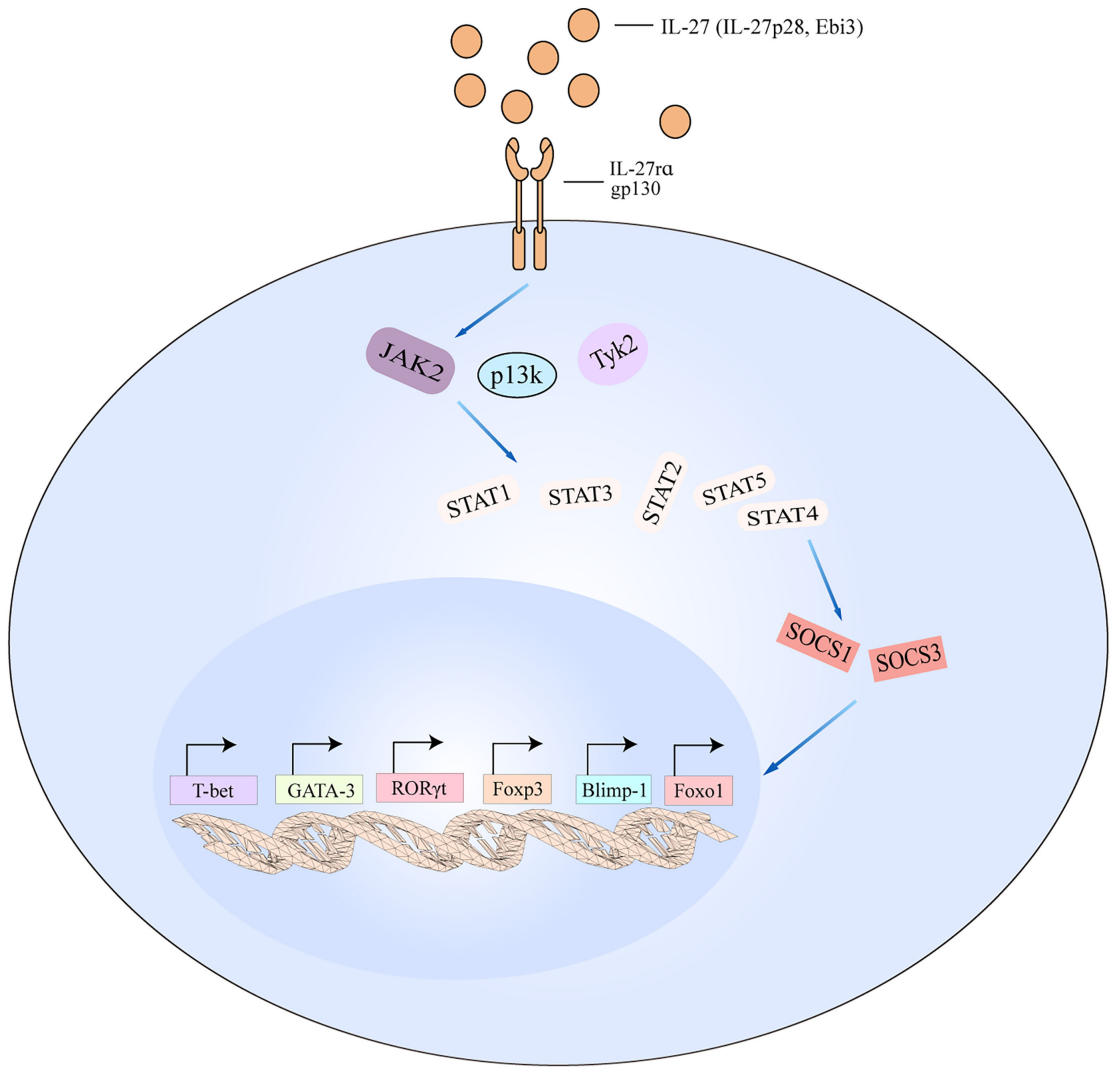
IL-27 signaling pathways in T cell. The schematic pathways listed are derived from the findings described in the text. Binding of IL-27 to its receptor results in the activation of JAK2, PI3K, and TyK2 signaling, which then activates downstream cascade including STAT1/STAT2/STAT3/STAT4/STAT5. The activated pathways interact with SOCS1 and SOCS3 to regulate the transcription of T-bet, GATA-3, RORγt, Foxp3, Blimp-1, and Foxo1. Blimp-1, B lymphocyte-induced maturation protein-1; Foxo1, forkhead box 1.

WT naive CD4^+^ T cells under Th1-polarizing conditions in the presence of IL-27 showed increased expression of Tim-3, IL-10, and NFIL3; elevated percentage of IFNγ^+^IL-10^+^ cells; and reduced expression of GM-CSF and receptor activator of nuclear factor-kappaB ligand (RANKL) (72, 74, 76, 81). NFIL3^−/−^CD4^+^ T cells under Th1-polarizing conditions in the presence of IL-27 led to the downregulated expression of Tim-3 and IL-10. NFIL3-overexpressed CD4^+^ T cells under Th1-polarizing conditions in the presence of IL-27 revealed an increase of IL-10 expression (72). STAT3^−/−^CD4^+^ T cells under Th1-polarizing conditions in the presence of IL-27 showed low expression of RANKL and IL-10 (81). Naive CD4^+^ T cells were primed with anti-CD3 and anti-CD28 in the presence of IL-27 under Th1-polarizing conditions, revealing increased expression of T-bet, ICAM-1, and LFA-1 (82). The addition of IL-12 or anti-ICAM-1 antibody or anti-LFA-1 antibody inhibited Th1 differentiation. Naive STAT1^−/−^CD4^+^ T cells stimulated with anti-CD3 and anti-CD28 in the presence of IL-27 did not induce T-bet expression (82). Thus, IL-27 drives Th1 differentiation by regulating NFIL3, STAT1, and STAT3 (**Figures 4**, **5**).

WT naive CD4^+^ T cells under Th2-polarizing conditions in the presence of IL-27 displayed low expression of RANKL and soluble RANKL (sRANKL) and high expression of IL-10 (81). STAT3^−/−^CD4^+^ T cells under Th2-polarizing conditions in the presence of IL-27 did not induce IL-10 expression, suggesting that IL-27 interacts with STAT3 and then regulates RANKL and IL-10 production in Th2 cells (81) (**Figure 4**).

WT CD4^+^ T cells stimulated with IL-27 under Th17-polarizing conditions showed low expression of IL-17, RANKL, and sRANKL, and activated STAT1 signaling (81, 83). Coculturing IL-27-treated CD45.2^+^CD4^+^ T cells with naive CD45.1^+^CD4^+^ T cells under Th17-polarizing conditions showed low expression of IL-17. Transferring IL-27-treated naive CD45.2^+^CD4^+^ T cells from OVA-TCR-transgenic (OT-II) mice with unprimed naive CD45.1^+^ OT-II T cells into WT mice showed a low percentage of CD45.1^+^IL-17A^+^ T cells. The addition of programmed cell death ligand-1 (PD-L1) neutralizing antibody abrogated IL-27-mediated inhibition of Th17 cell differentiation (83). CD4^+^ T cells from STAT3^−/−^ mice treated with IL-27 under Th17-polarizing conditions showed low expression of RANKL, and RANKL was significantly reduced from WT mice treated with IL-27 under Th17-polarizing conditions (81). When naive CD4^+^ T cells were activated with anti-CD3, anti-CD28, TGF-β, and IL-6, there was increased expression of IL-17A, IL-17F, and IL-23R (84). The addition of IL-27 inhibited the expression of Th17-related cytokines and RORγt expression. CD4^+^ T cells from STAT1^−/−^ mice treated with IL-27 did not suppress the expression of IL-17A and RORγt (84). Similarly, CD4^+^CD25^−^ T cells treated with TGF-β+IL-6 in the presence of IL-27 did not develop into Th17 cells (85). Collectively, IL-27 inhibits Th17 development through STAT1- and STAT3-mediated suppression of RORγt (**Figure 4**).

Mesenteric lymph nodes are stimulated with anti-CD3 and anti-CD28 in the presence of IL-6, showing more Tfh cell differentiation and high expression of CXCR5, c-Maf, and T cell factor 1 (TCF1) (86). The addition of IL-21 to IL-6-induced Tfh cells promoted the production of TCF1 and KLF2, and the addition of IL-2 to IL-6-induced Tfh cells increased the expression of Foxo1 and T-bet and activated STAT5. In contrast, the addition of IL-27 decreased the percentage of Tfh cells and CXC chemokine receptor type 5 (CXCR5) expression, but upregulated the expression of Foxo1, T-bet, and STAT5 in Tfh cells than effects of IL-2 on Tfh cells, indicating that IL-27 inhibits Tfh cell differentiation (86) (**Figure 4**).

Total CD8^+^ T cells from adeno-associated viral vector (AAV)-IL-27-treated WT mice showed high expression of T-bet, Eomes, and Blimp-1, especially in memory CD8^+^ T cells (77). naïve CD8^+^ T cells from WT mice stimulated with IL-27 showed low expression of GM-CSF and high expression of IL-10, T-bet, IL-12Rβ2, granzyme B, and perforin, and activated STAT1, STAT2, STAT3, STAT4, and STAT5 (74, 76, 87). The addition of JAK2 and Tyk2 inhibitors upregulated the expression of GM-CSF (74). Under Th0-polarizing conditions, IL-27 induced IL-10 and IFNγ expression in WT CD8^+^ T cells, and IL-27 upregulated IL-10 production in CD8^+^IFNγ^+^ T cells under Tc0- and Tc1-polarizing conditions (76). In contrast, STAT1^−/−^CD8^+^ T cells stimulated with IL-27 did not increase the production of IL-10, T-bet, granzyme B, perforin, and IL-12Rβ2 (76, 87). Moreover, WT CD44^high^SLAMF6^+^CXCR6^−^CD8^+^ cells simulated with IL-27 led to an elevated proportion of CD44^high^SLAMF6^+^CXCR6^−^CD8^+^ cells, especially the TCF1^−^CXCR6^+^ phenotype, and IL-27-stimulated CD44^low^ cells showed low expression of TCF1 and high expression of CXCR6 (88). These findings indicate that IL-27 acts on WT CD8^+^ T cells in a JAK2- and STAT1-dependent manner and promotes the generation of cytotoxic T lymphocyte (CTL) with an elevated expression of granzyme B.

Overexpression of IL-27 (IL-27Tg) in WT mice resulted in an increase of PD-L1, lymphocyte-activation gene-3 (LAG-3), T cell immunoglobulin, and ITIM domain (TIGIT), and Tim-3 expression in T cells (89). IL-27 transgenic mice showed a high percentage of total CD8^+^ T cells in peritoneal exudate cells (PECs), and a high percentage of CD8^+^ and CD4^+^ T cells in the spleen that produced IFNγ (90). There was a low percentage of CD4^+^ T cells that produced IL-2 in the spleen from IL-27Tg mice (90). Thus, IL-27Tg mice showed a high percentage of total CD4^+^ and CD8^+^ T cells and a low proportion of Th cells.

There were lower percentages of CD4^+^IFNγ^+^ T cells, a higher number of CD4^+^IL-17A^+^ T cells, and a higher expression of IL-17 in the lungs of IL-27rα^−/−^ mice as compared with that in WT mice (44). After immunizing with OVA/CFA, CD4^+^ T cells from IL-27rα^−/−^ mice showed reduced expression of IL-21 (68). Similarly, immunizing IL-27rα^−/−^ mice with TNP-OVA in Freund’s adjuvant reduced the percentage of CD4^+^PD1^+^CXCR5^+^ T cells in the spleen and draining lymph nodes (dLNs). IL-27rα^−/−^ mice revealed a reduction in CD4^+^ T cells with a CXCR5^+^, PD1^+^, ICOS^+^, CCR7^lo^, CD62L^lo^, and CD127^lo^ cell phenotype (a phenotype of Tfh cell). The percentage of annexin V^+^ cells in the PD1^+^CXCR5^+^ Tfh cells was much higher in the spleen and LN from IL-27rα^−/−^ mice compared with WT mice (68). T cells from mice with IL-27rα^−/−^CD4^+^ T cells were stimulated with IL-27, showing increased phosphorylation expression of STAT1, STAT3, STAT4, and STAT5 (91). T cells from mice with IL-27rα^+/+^CD4^+^ T cells were stimulated with IL-27 in the presence of anti-CD3 and anti-CD28 antibodies, showing increased expression of T-bet, whereas there was no increase of T-bet in IL-27rα^−/−^CD4^+^ cells. IL-27rα^+/+^CD4^+^ T cells treated with IL-27 under Th1-polarizing conditions showed a higher expression of IL-12Rβ2 and a lower expression of GATA-3. However, IL-27 treatment did not inhibit GATA-3 expression in STAT1^−/−^CD4^+^ T cells, suggesting that IL-27-mediated GATA-3 inhibition depends on STAT1 activation (91). IL-27rα^−/−^ mice failed to expand CD8^+^CXCR5^+^ T cells and showed a lower number of Tfh and CD8^+^ T cells after IFNα/β receptor 1 (IFNAR1) treatment (92). There was reduced expression of stem cell antigen-1 (Sca-1)/Ly6A in CD4^+^ and CD8^+^ T cells from IL-27rα^−/−^ and Ebi3^−/−^ mice (77). CD8^+^CXCR5^+^ T-cell expansion was inhibited in Ebi3^−/−^ mice, and Ebi3^−/−^ mice treated with IFNAR1 revealed an increase in percentage of CD8^+^CXCR5^+^ T cells in inguinal LNs, indicating that IL-27 was required for IFNAR1-induced CD8^+^CXCR5^+^ T-cell expansion (92).

WT mice infected with *herpes simplex virus type 1* (*HSV-1*) in the presence of anti-IL-27 antibody showed less CD4^+^ T-cell infiltration in the spleen and dLNs (93). WT mice infected with *respiratory syncytial virus* (*RSV*) and then administered with IL-27 neutralizing antibody revealed elevated virus-specific CD8^+^ and CD4^+^ T cells in the lung, increased IFNγ and TNFα production, and reduced expression of IL-10 in the airways (94). WT mice infected with *strongyloides stercoralis* (*SS*) in the presence of anti-IL-27 antibody showed higher frequencies of Th1, Th2, Th9, Th17, and Th22 cells; more CD8^+^ cells expressing IFNγ, IL-4, IL-5, IL-13, IL-9, IL-17, and IL-22; and high expression of IFNγ, IL-5, IL-9, IL-17, and IL-22 (95). IL-27p28-overexpressing mice infected with *Toxoplasma gondii* led to increased serum levels of IFNγ and increased percentage of CD4^+^IFNγ^+^ and CD8^+^IFNγ^+^ T cells (96). Similarly, IL-27rα^−/−^ mice infected with *Leishmania donovani* showed increased numbers of total CD4^+^ and Th1 cells, elevated serum levels of IFNγ and TNFα, and lower numbers of PEPCK^+^ Tr1 cells in the liver (97). IL-27rα^−/−^ total CD4^+^ T and Th1 cells showed increased mitochondrial mass and membrane potential, larger size, and more granularity after infecting with *L. donovani*, suggesting that IL-27 signaling inhibited mitochondrial changes in CD4^+^ T cells during *L. donovani* infection (97). IL-27rα^−/−^ mice infected with *sendai virus* (*SeV*) revealed reduced frequency of CD8^+^ T cells, CD4^+^IL-10^+^, and CD4^+^IFNγ^+^IL-10^+^ T cells and an increased number of CD4^+^IL-17A^+^ and CD4^+^IL-13^+^ T cells in the lung, indicating that after *SeV* infection, IL-27 limited Th2 and Th17 responses (57). IL-27rα^−/−^ mice infected with *Trypanosoma cruzi* showed high expression of IL-17 (98). Collectively, IL-27 differently regulates T-cell function according to distinct infection.

A smoking mouse model of emphysema reported high expression of IL-27, and strong CD8^+^IFNγ^+^ T-cell response (99). IL-27 stimulation with CD8^+^IFNγ^+^ T cells inhibited differentiation of CD8^+^IFNγ^+^ T cells, and injection of IL-27 into the mice attenuated CD8^+^IFNγ^+^ T-cell response after cigarette smoke exposure (99). Injection of IL-27 into the OVA-induced asthma mouse model showed a high percentage of Th1 and total memory T cells and lower expression of Th2-related cytokines IL-4, IL-5, and IL-13 (100). Interestingly, IL-27rα^−/−^ were induced to experimental allergic conjunctivitis (EAC), showing stronger Th2- and Th17-dominant responses in conjunctiva and cervical lymph nodes, evidenced by the high expression of IL-4, IL-5, IL-13, GATA-3, and IL-17 and the higher percentage of IL-17A^+^ cells in conjunctival stroma. In contrast, IFNγ and IL-10 expression was reduced in IL-27rα^−/−^ EAC mice, demonstrating that IL-27 signaling deficiency develops Th17- and Th2-dominant inflammation under disease conditions (101).

#### 3.1.3 IL-27 promotes Treg cell differentiation and function in humans, but inhibits Treg cell function in mouse models

Regulatory T (Treg) cell is a kind of Th cell that mainly displays a regulatory role in immune responses. To date, findings about the role of IL-27 in Treg cell is partly different either in humans or in mouse models. For instance, naive CD4+ T cells from healthy controls stimulated with IL-27 under iTreg-polarizing conditions showed an upregulated percentage of Foxp3+ cells and induced STAT1 phosphorylation (102). Coculturing IL-27-treated iTreg cells with autologous CD4+ T cells showed less proliferation of CD4+ T cells. Activated STAT1 recognized and bound to STAT binding sites in the Foxp3 gene promoter. IL-27 stimulation increased the transactivation of the Foxp3 gene promoter. In IL-27-treated iTreg cells, histone H4 molecule was acetylated in the Foxp3 gene promoter, indicating that IL-27 regulates Treg cell function via STAT1 signaling in humans (102). As compared to effects of IL-27 in humans, naive CD4+ T cells and CD4^+^CD25^−^ T cells from WT mice stimulated with TGF-β induced Foxp3+ Treg cell differentiation and CD25 and anti-cytotoxic T-lymphocyte Antigen-4 (CTLA-4) expression, and downregulated IL-2 and TNFα expression (85, 103). The addition of IL-27 potently inhibited the development of Treg cells, downregulated CD25 and CTLA-4 expression, upregulated TNFα expression, and induced the phosphorylation of STAT3, whereas the addition of sIL-27p28-Fc led to increased Treg cell development (85, 103). When CD4^+^CD25^−^ T cells were cocultured with IL-27-treated Treg cells, there was proliferation of CD4^+^CD25^−^ T cells (85, 103). STAT3^−/−^CD4^+^ T cells treated with TGF-β in the presence of IL-27 showed high expression of Foxp3 (103). All these indicated that IL-27 inhibits the development of Treg cells via STAT3. It is interesting that IL-27 shows an inhibitive effect on Treg cells based on the above findings. IL-27-mediated reduction of Foxp3+ Treg cells was concentration dependent and correlated with study time points. That is, IL-27 regulates early development rather than late differentiation of Treg cells (85) (**Figure 4**). More interestingly, IL-27 may promote effects of Treg cell by regulating STAT1 in humans and inhibit effects of Treg cell by regulating STAT3 in mouse models. However, this needs to be demonstrated in the future with functional studies. Furthermore, IL-27rα^−/−^ and Treg-specific IL-27rα^−/−^ mice were injected with cockroach antigen, showing severe airway inflammation, whereas IL-27 treatment showed little effects on reducing the inflammatory responses (104). However, IL-27-induced treatment was restored after transferring WT Treg cells but not transferring Treg cells deficient in Lag3, a molecule induced by IL-27 in Treg cells. The findings suggested that IL-27 targets Foxp3^+^ Treg cells to mediate anti-inflammatory effects during experimental allergic airway inflammation in mouse models (104). In mouse models with experimental autoimmune encephalomyelitis (EAE), systemic delivery of IL-27 effectively prevented development of EAE, whereas systemic delivery of IL-27 in EAE mice without Treg cells did not inhibit neuroinflammation (105). Similarly, transferring Treg cells deficient in IL-27rα or Lag3 into EAE mice did not inhibit EAE development. These findings were observed in mice with IL-27rα^−/−^ Treg cells. When the mice were subcutaneously immunized with MOG35-55 peptide, there were much numbers of CD4+IFNγ+, CD4+IL-17A+ T cells in the CNS tissue. (106), indicating that IL-27 attenuated autoimmune neuroinflammation via Treg cells. In colitis mouse models, transferring WT Treg cells was capable of suppressing colitogenic T-cell expansion and inflammatory cytokines IL-17A and IFNγ expression (107). However, transferring IL-27rα^−/−^ Treg cells into colitis mouse models did not inhibit inflammatory T-cell responses. In mouse models with graft-versus-host disease (GvHD), transferring control Treg cells rescued half of the mice from lethal disease, whereas transferring IL-27 pre-stimulated Treg cells rescued 90% of the mice from lethal disease, and alleviated GvHD scores (108). *Bacteroides fragilis* stimulated the generation of IL-27 in WT mice BMDCs (109). WT BMDCs were stimulated with *B. fragilis* and then were co-cultured with WT CD4^+^ T cells, showing induced CD4^+^Foxp3^+^ Treg cells. Foxp3^+^ Treg cells stimulated with IL-27 also showed high expression of IL-10, and treatment with commensal bacteria induced the generation of IL-27 and IL-10 in BMDCs and Treg cells, revealing that IL-27 may be directed on Treg cells to promote tolerogenic function (109). Interestingly, IL-27 was able to promote the expression of T-bet and CXCR3 in Treg cells (110). After infecting with *T. gondii*, WT mice showed severe inflammatory T-cell responses, whereas the injection of IL-27 significantly limited T-cell responses at mucosal sites. When transferring Treg cells into IL-27rα^−/−^ mice infected with *T. gondii*, there was alleviated pathology. Together, the findings indicated that IL-27 is a key cytokine that promotes Treg cell function in different pathogenic conditions. As discussed above, IL-27 is able to inhibit Treg cells function in mice under normal conditions, while IL-27 may promote Treg cell function under pathogenic conditions in mouse models. Nevertheless, a clear role of IL-27 in Treg cells in mice needs further discussion.

### 3.2 IL-27 promotes differentiation of B cell and maintains antibody production

The total CD19^+^ B cells from patients with chronic lymphocyte leukemia (CLL) stimulated with IL‐27 showed elevated apoptosis of B cells and downregulated proliferation of B cells, whereas IL-27 stimulation on CD19^+^ B cells from healthy controls did not promote B-cell apoptosis (111). Naive CD20^+^CD38^−^CD27^−^ B cells from healthy controls stimulated with anti-μ Ab induced a few cell divisions, and the addition of IL-27 promoted naive B-cell division that left the G0/G1 stage and entered the S phase (60). Administration of IL-27 in naive B cells upregulated the expression of Pim-1, cyclin D2, cyclin D3, and cyclin A. In contrast, Pim-1 inhibitor reversed anti-μ Ab- and IL-27-mediated effects, evidenced by decreased expression of cyclin A, cyclin D2, and cyclin D3 (60). Naive CD19^+^CD27^−^ B cells were stimulated with anti-CD40+IL-27, showing an elevated expression of IgG1 (112). Interestingly, IL-27 stimulation with naive CD19^+^CD27^−^sIgD^+^sIgG^−^ B cells did not induce IgE production. When naive CD19^+^CD27^−^sIgD^+^sIgG^−^ B cells were primed with IL-4 and then were stimulated with IL-27, there was increased expression of IgE (112). Incubation of naive CD20^+^CD38^−^CD27^−^ or total CD19^+^ or memory CD20^+^CD38^-^CD27^+^ B cells with IL-27 led to the activation of STAT1 and STAT3 (113). When total CD19^+^ B cells were stimulated with anti-CD40 Ab, or anti-μ Ab in the presence of IL-27, there was increased T-bet, IL-12Rβ1, IL-12Rβ2, CD54, CD86, and CD95 expression and increased B-cell proliferation (113). Moreover, naive CD10^−^CD20^+^CD27^−^IgG^-^ B cells stimulated with CD40L and anti-Ig Ab in the presence IL-27 showed a high percentage of cells acquiring a CD20^+^CD38^+^ phenotype and increased expression of CD95 (114). Splenic B cells from WT mice were stimulated with CD40L+IL-27, revealing an increase in the percentage of B cells devoid of sIgD and high levels of CD38, and reflecting differentiation into a CD38^high^ plasma cell phenotype (112). IL-27-induced CD38^high^ B cells showed low levels of CD20 and high levels of CD27 (112). Splenic B cells from WT mice were stimulated with IL-27, displaying increased T-bet expression (115). T-bet expression was increased in splenic B cells stimulated with anti-CD40+LPS as well. IgG2a generation was increased by incubating B cells with IL-27+LPS. However, splenic B cells stimulated with IL-27 in the presence of IL-4 did not induce IgG1 class switching, and did not induce T-bet expression. In STAT1^−/−^ splenic B cells, IL-27 stimulation barely induced T-bet expression and downregulated IgG2a generation. Similarly, in T-bet^−/−^ splenic B cells, IL-27+anti-CD40, or IL-27+LPS stimulation inhibited IgG2a generation (115). IL-27rα^−/−^ mice had a defect in generating regulatory B (Breg) cells in response to BCR activation (111). In IL-27p28^−/−^ mice, there was a low percentage of Breg cells. The addition of IL-27 to IL-27rα^−/−^ B cells or IL-27p28^−/−^ B cells upregulated IL-27 expression and promoted the expansion of IL-27-producing B cells (116). There was a lower expression of IL-27 in total B cells and plasma B cells derived from MB1-Cre^+/−^/IL-27p28^−/−^ mice compared with that in IL-27p28^−/−^ mice (117). MB1-Cre^+/−^/IL-27p28^−/−^ mice infected with *lymphocytic choriomeningitis virus* (*LCMV*) showed less virus specific I-A^b^ GP_67-77_ tetramer^+^ CD4^+^ T cells and IFNγ-producing CD4^+^ T cells and Tfh cells (117). Tfh cells from B cell-specific IL-27p28^−/−^ mice stimulated with LCMV GP_61-80_ peptide revealed less IFNγ^+^IL-21^+^ cells and lower IgG2a/2c expression (117). All these suggest that IL-27 induces the differentiation of B cells and maintains Tfh function, antibody production, and clearance of a persistent virus.

## 4 Association of IL-27 and inflammatory autoimmune diseases

### 4.1 Both elevated and reduced expression of IL-27 in systemic lupus erythematosus and IL-27 both promotes and inhibits lupus development

Systemic lupus erythematosus (SLE) is a chronic and complex autoimmune disease. In Polish patients with SLE (either treated with corticosteroid or not), serum levels of IL-27 were not correlated with SLE disease activity index (SLEDAI) score and anti-dsDNA, C3, and C4 levels (118). This was confirmed in patients with lupus nephritis (LN), where serum levels of IL-27 were not related to SLEDAI score and anti-dsDNA, C3, and C4 levels (118). In Southern Chinese patients with SLE (treated with corticosteroid) or Brazilian patients with SLE (treated with immunosuppressive agents), serum levels of IL-27 were lower than those in healthy controls, which were not related to SLEDAI score (119, 120) (**Table 1**). Patients with LN reported reduced serum levels of IL-27 as compared to that in patients with SLE without LN (120). In contrast, levels of IL-27 in serum and urine from Northern Chinese patients with SLE (without treatment), or LN (without treatment), were increased as compared to those in healthy controls (121). Urine levels of IL-27 were related to the renal SLE disease activity index score and 24-h urinary protein levels. Interestingly, patients with LN revealed much higher urine levels of IL-27 after immunosuppressive treatment (121). In PBMCs of North American patients with SLE (without treatment), expression of IL-27 was increased compared to that in healthy controls (122). When the patients with SLE were divided into two groups, patients with high type 1 interferon (IFN) signature and patients with type 1 IFN signature similar to the healthy controls, expression of IL-27 was elevated in the patients with high type 1 IFN signature, whereas expression of IL-27 was comparable between patients with type 1 IFN signature similar to the healthy controls and healthy controls, suggesting that the type 1 IFN signature was related to higher levels of IL-27 (122). With respect to IL-27 genetic mutation in patients with SLE, rs153109 polymorphism was not related to Egyptian and Polish patients with SLE (161, 162), and rs181206 polymorphism was not related to Polish patients with SLE (162). Rs17855750 genotypes TT, TG, TG+GG, and allele T were related to SLE risk in Egyptian patients (161); haplotype CG [rs181206 (C)+rs153109 (G)] was related to a higher risk of SLE in Polish patients; and haplotype TG [rs181206 (T)+rs153109 (G)] was negatively related to SLE risk in Polish patients (162) (**Table 2**). Several reasons may correlate with the above distinct findings, such as treatment and ethnicity.

**Table 1:** Expression of IL-27 in inflammatory autoimmune diseases.

Disease	Sample	Expression	Treatment	Ethnicity	Reference
SLE	Serum	Reduced^a^	Steroids, Antimalarial agents, Azathioprine, Micophenolate mofetil, Thalidomide	Brazilian	(119)
	Serum	Reduced^a^	Corticosteroid	Southern Chinese	(120)
	Serum, urine	Increased^a^	None	Northern Chinese	(121)
	PBMC	Increased^a^	None	North American	(122)
RA	Plasmablast, serum, Breg cell	Increased^a^	None	Northern Chinese	(123)
	Serum	Increased^a^	None	Southern Chinese, Northern Chinese	(124, 125)
	Plasma	Increased^a^	None	Southern Chinese	(126)
	Synovium, synovial fluid	Increased^a^	None	Japanese	(127)
	Synovitis	Reduced^a^	Methotrexate	UK	(128)
SS	Serum	Increased^a^	Unknown	Northern Chinese	(129)
	Serum, salivary glands	Increased^a^	Unknown	UK	(130)
	PBMC, serum	Reduced^a^	Prednisone, Hydroxychloroquine, Cyclophosphamine, Leflunomide	Southern Chinese	(131)
	Serum	Reduced^b^	None	–	(131)
	Plasma	Reduced^a^	Unknown	Northern Chinese	(132)
BD	Serum	Reduced^a^	Unknown	Turkish	(133)
	Serum, PBMC	Reduced^a^	None	Southern Chinese	(134)
	Serum	Increased^a^	Prednisone	Southern Chinese	(135)
	Serum	Increased^a^	None	Iranian	(136)
IBD	PBMC	Normal^a^	5-aminosalicylic acid, Prednisone, Azathioprine (AZA), Infliximab	Iranian	(137)
	Colonic biopsy	Increased^a^	Mesalazine, Azathioprine, Prednisone, Mercaptopurine	Mexican	(138)
	Colonic mucosa	Increased^a^	Steroids, Azathioprine	German	(139)
	Intestinal mucosa	Increased^a^	Mesalazine, Corticosteroids, Methotrexate, Azathioprine	German	(140)
	Serum, stool, moDC	Increased^a^	Unknown	Southern Chinese	(141)
	Serum, cecum	Increased^b^	None	–	(141)
MS	Serum	Reduced^a^	None	Romanian, Northern Chinese, Iranian	(142–144)
	Serum, cerebrospinal fluid	Increased^a^	Corticoids	Swiss	(145)
	Plasma	Increased^a^	INFβ	Iranian	(146)
	Serum, cerebrospinal fluid	Increased^a^	Unknown	Canadian	(147)
	Serum	Increased^b^	Unknown	–	(148)
	Brain tissue	Increased^a^	Unknown	Canadian	(149)
Psoriasis	Serum	Increased^a^	None	Brazilian	(150)
	Serum	Increased^a^	Steroids, Vitamin D3	Japanese	(151)
	Serum, epidermis, dermis, PBMC	Reduced^a^	None	Southern Chinese	(152)
SSc	Serum, B cell, CD4^+^ T cell	Increased^a^	None	Japanese	(153)
T1D	Serum	Normal^a^	None	Polish	(154)
	Serum	Reduced^a^	Insulin	Iranian	(155)
	Plasma	Increased^a^	Insulin	Estonian	(156)
	Treg cell	Reduced^a^	Insulin	Polish	(157)
VKH	Serum, PBMC	Reduced^a^	None	Southern Chinese	(158)
	Serum	Increased^a^	None	Southern Chinese	(159)
AS	Serum, synovial fluid	Increased^a^	Unknown	Northern Chinese	(160)

SLE, systemic lupus erythematosus; RA, rheumatoid arthritis; SS, Sjogren syndrome; BD, Behcet’s disease; IBD, inflammatory bowel disease; MS, multiple sclerosis; SSc, systemic sclerosis; T1D, type 1 diabetes; VKH, Vogt–Koyanagi–Harada; AS, ankylosing spondylitis; PBMC, peripheral blood mononuclear cells; Breg cell, regulatory B cell; moDC, monocyte-induced dendritic cell; Treg cell, regulatory T cell.

a Human.

b Mice.

**Table 2:** Association of IL-27 gene polymorphisms with inflammatory autoimmune disease.

Disease	Polymorphism	Significant association	Ethnicity	Reference
SLE	rs153109	No	Egyptian	(161)
	rs17855750	Yes	Egyptian	(161)
	rs181206	No	Polish	(162)
RA	rs153109, rs17855750, rs181206	No	Chinese	(163)
	rs153109, rs181206	Yes	Polish	(164)
BD	rs153109	Yes	Iranian	(136, 165)
	rs181206	No	Iranian	(165)
IBD	rs153106	Yes	Chinese, Korean	(166, 167)
	rs181206, rs17855750	No	Korean	(167)
	rs17855750	Yes	Mexico	(168)
	rs428253, rs4740, rs4905	No	Mexico	(168)
MS	rs181206, rs153019	Yes	Romania	(142, 169)
T1D	rs153109, rs34833, rs26528, rs17855750, rs18126, rs40837	No	Brazilian	(170)
VKH	rs4788084	No	Chinese	(171)

SLE, systemic lupus erythematosus; RA, rheumatoid arthritis; BD, Behcet’s disease; IBD, inflammatory bowel disease; MS, multiple sclerosis; T1D, type 1 diabetes.

Transferring bone marrow cells from lupus-prone BXD2 mice into apolipoprotein E-deficient (Apoe^−/−^) mice (ApoE^BXD2^) in the presence of high-fat diet (HFD)-induced hyperlipidemia increased levels of IgG, IgG2c, anti-dsDNA, rheumatoid factor (RF), and severe glomerulonephritis (including expanded double layers around glomerulus, crescentic tubules, and deposition of immune complex) (172). HFD-fed Ebi3^−/−^Apoe^−/−^ mice revealed reduced frequency of germinal center (GC) B cells and CXCR3^+^CCR6^−^ Tfh cells, suggesting that an atherogenic environment in Apoe^−/−^ mice resulted in autoimmune lupus, where IL-27 is important for Tfh cells and GC reactions (172). In IL-27rα^−/−^Roquin^san/san^ mice, there was reduced kidney pathology, such as reduced glomerular and tubulointerstitial nephritis and vasculitis, and there was ameliorated splenomegaly, a lower number of CD4^+^CXCR5^+^PD1^+^ Tfh cells and B220^+^GL7^+^CD38^lo^ GC B cells, and a higher percentage of CD138^+^ plasma cells compared with IL-27rα^+/+^Roquin^san/san^ mice (114). Similarly, IL-27rα^−/−^Roquin^san/san^ mice had an elevated percentage of GC B cells switching to IgG1 and fewer cells switching to IgG2a(c), and these mice showed a lower number of CD4^+^ and CD44^+^ cells, and lower ICOS levels, demonstrating that IL-27 promoted GC B-cell activity and potentiated lupus in Sanroque mice (114). Pristane-treated IL-27rα^−/−^ mice reported a reduced number of Tfh and GC B cells, along with low levels of anti-nuclear antibody (ANA) and anti-dsDNA antibody, a low renal histopathology score, and reduced immune complex deposition in the kidney as compared to control mice (68). These findings were different from spontaneous MRL/lpr lupus mice (173, 174). In IL-27rα^−/−^ MRL/lpr mice, the mice had a higher expression of proteinuria, diffuse thickening of peripheral capillary walls, with spikelike alterations of basement membranes, and widespread discrete, granular deposition localized to the glomerular capillary walls (174). IL-27rα^−/−^ MRL/lpr mice developed skin inflammation characterized by erythema, crust formation, erosion, alopecia and lichenification on the dorsal, periocular, nasal, and ear regions (173). Epidermal hyperplasia and dermal infiltration of mononuclear cells were revealed in IL-27rα^−/−^ MRL/lpr mice (173). Moreover, IL-27rα transgenic MRL/lpr mice indicated longer survival than control mice, and the transgenic mice had lower blood urea nitrogen (BUN) and urinary protein:creatinine ratio, and splenomegaly and lymphadenopathy were reduced (175). There were reduced inflammatory cell infiltration, glomerular sclerosis, mesangial proliferation, and crescent formation in kidney and a lower score of glomerular proliferative activity in IL-27rα transgenic mice, and the transgenic mice showed less IgG deposition and mesangial lesions, and low levels of ANA, anti-dsDNA antibody, and total IgG and IgG2a. Expression of IFNγ, IL-4, and IL-12b in splenic CD4^+^ T cells was reduced, and percentages of CD4^+^ and CD8^+^ T cells and CD3^+^B220^+^CD4^−^CD8^−^ T cells were downregulated in the transgenic mice (175), suggesting that IL-27rα inhibited the development of autoimmune nephritis in MRL/lpr mice. Considering the differences between IL-27rα^−/−^Roquin^san/san^ mice, pristane-treated IL-27rα^−/−^ mice, and IL-27rα^−/−^ MRL/lpr mice, several aspects may correlate with different results of lupus development, such as different mouse models and differences in the experimental setup. It is notable that in Roquin^san/san^ mice and pristane-treated mice, GCs drove disease, and the deficiency of IL-27rα led to disease severity, highlighting the role of IL-27 in promoting high-affinity Ab production in autoimmune lupus (68, 114). In contrast, imbalance of the Th1/Th2 immune responses contributed to the phenotype of glomerulonephritis and skin lesions in MRL/lpr mice, by which IL-27rα gene disruption caused skewing of immune responses toward Th2 (173, 174).

### 4.2 Increased expression of IL-27 in rheumatoid arthritis, which both promotes and inhibits arthritis development

Rheumatoid arthritis (RA) is an inflammatory autoimmune disease characterized by symmetrical small joint injury in patients. Patients with RA had elevated frequencies of CD19^+^CD27^+^CD38^high^ plasmablasts and CD19^+^CD138^+^ plasma cells, and much higher expression of erythrocyte sedimentation rate (ESR), serum IgM, and IgG as compared to those in controls (123). Serum levels of IL-27 were elevated in patients with RA as well, which were related to disease activity score in 28 joints (DAS28), frequencies of plasma cells, and the levels of autoantibodies (123–125) (**Table 1**). Serum levels of IL-27 were higher in patients with RA with interstitial lung disease (ILD) than that in patients without ILD (125). Elevated plasma levels of IL-27 were observed in patients with RA compared to that in controls (126). Similarly, IL-27 was elevated in RA synovial fluid than in osteoarthritis (OA) synovial fluid, and there were more CD14^+^IL-27^+^ cells in RA synovium but rarely in patients with OA (127). By contrast, immunosuppressive treatment was able to downregulate IL-27 expression in patients with RA (124, 128). For IL-27 gene polymorphisms, a study involving a Chinese Han population reported that rs153109, rs17855750, and rs181206 polymorphisms did not correlate with RA risk, whereas frequency of haplotype GTC [rs153109 (G) + rs17855750 (T) + rs181206 (C)] was higher in patients with RA, and frequency of haplotype GTT [rs153109 (G) + rs17855750 (T) + rs181206 (T)] was lower in patients with RA than that in controls, respectively (163) (**Table 2**). Another study in Polish patients with RA found that frequencies of rs153109 GG genotype and G allele, haplotype CG [rs181206 (C) + rs153109 (G)] were higher in patients with RA, and frequency of haplotype CA [rs181206 (C) + rs153109 (A)] was lower in patients with RA than that in controls, respectively (164). IL-27 rs181206 polymorphism correlated with HAQ score and ESR levels, and patients with RA carrying the rs153109 G allele showed advanced disease compared to patients carrying the A allele. Thus, the expression of IL-27 was elevated in patients with RA, and IL-27 gene haplotypes were related to risk of RA.

Patients with RA had reduced frequencies of peripheral CD19^+^CD27^+^CD24^high^ Breg cells, and the cells were more proliferated and expressed high expression of activation markers CD80 and CD86 (123). B cells from patients with RA showed high expression of activated mTOR, and the serum-treated B cells of patients with RA revealed higher expression of mTOR. The addition of anti-IL-27 neutralizing antibody inhibited B-cell dysfunction, indicating that IL-27 may promote peripheral B-cell dysfunction in patients with RA by activating mTOR signaling (123). RA fibroblast-like synoviocytes (RA-FLSs) treated with IL-27 induced the activation of STAT1 and increased the surface expression of intercellular adhesion molecule (ICAM)-1, vascular cell adhesion molecule (VCAM)-1, IL-6, CCL2, CXCL9, CXCL10, and matrix metalloproteinase-1 (MMP-1). RA-FLSs treated with IL-27 and TNFα further upregulated the expression of ICAM-1, VCAM-1, CXCL9, and CXCL10 (126), indicating that IL-27 combined with TNFα may contribute to inflammatory component production. WT rats injected with complete Freund’s adjuvant had severe clinical features of arthritis, and the addition of IL-27 antagonist downregulated villous hyperplasia, infiltration of the inflammatory cells, pannus formation, and angiogenesis (176, 177). IL-27rα^+/+^ mice immunized with proteoglycan (PG) had characteristics of arthritis, and development of arthritis was suppressed in IL-27rα^−/−^ mice (178). IL-27rα^−/−^ mice treated with PG had less mononuclear and polymorphonuclear cell infiltration in the joints, less edema of the synovial and periarticular tissues, less cartilage erosion, and less disintegrating chondrocytes in the remaining layer of the articular surface (178). There was lower expression of PG-specific IgG2a, TNFα, IL-6, IFNγ, IL-17, and IL-1β in IL-27rα^−/−^ mice, and T cells from peritoneal cavity generated more IFNγ than spleen T cells, indicating that PG-specific T cells migrated to the peritoneal cavity (178). Moreover, the IL-27p28 subunit injected into adjuvant-induced arthritis rats amplified autoantibodies’ production, whereas administration of a targeted DNA vaccine encoding IL-27p28 into the arthritis rats revealed the downregulated form of the disease, less paw swelling, and reduction in inflammatory mononuclear cell infiltration in the synovial membrane; the thickness of the synovial lining, cartilage destruction, expression of collagen type II, and proliferation of Ag-specific T cells were decreased (179). Therefore, IL-27 may promote arthritis development.

Since the pro-inflammatory role of IL-27 was widely discussed in arthritis models, it is recognized that numerous studies indicated the anti-inflammatory effects of IL-27 in arthritis development (180–183). Mononuclear cells (MNCs) from RA patients’ FLSs stimulated with TNFα or IL-17 induced expression of IL-27, IL-6, and CCL20; however, the addition of IL-27 to the cells inhibited IL-6 generation induced by TNFα or IL-17 (127). Human CD14^+^ cells cultured with M-CSF+RANKL promoted differentiation of TRAP^+^ cells and induced NF-ATc1 expression, whereas administration of IL-27 suppressed osteoclastogenesis, and decreased the expression of cathepsin K and integrin β3 (184). RA patients’ memory CD4^+^ T cells cultured with IL-27 downregulated differentiation of Th17 cells and secretion of IL-17 (185). Similarly, naive CD4^+^ T cells from collagen-induced arthritis (CIA) mice treated under Th1-, Th2-, or Th17-polarizing conditions upregulated RANKL expression, whereas the addition of IL-27 suppressed RANKL expression and upregulated IL-10 expression (186). Murine bone marrow-derived osteoclast precursors stimulated with RANKL further led to the formation of TRAP^+^ cells (184). IL-27 administration suppressed osteoclast formation, and reduced the number of TRAP^+^ cells (184). Furthermore, B10.RIII mice transferred *IL-23* gene induced significant arthritis, including increased disease incidence, disease severity score (evidenced by increased paw swelling and synovial hyperplasia in ankle joints), and neutrophil infiltration into the joints (8). The addition of IL-27 into IL-23-induced arthritis mice ameliorated disease incidence and disease severity, inhibited neutrophil proliferation and γδ T-cell accumulation, and resulted in reduction in serum levels of IL-6, IL-17, and IgG2a, and percentage of cells expressing IL-17 and IFNγ (180, 181). The findings suggested that IL-27 may inhibit the effects of TNFα, IL-17, RANKL, and IL-23, and then suppress arthritis development. In CIA mice, treatment with IL-27-Fc ameliorated arthritis severity and incidence as well, shown as less inflammation; less cartilage damage; reduced levels of CII-specific IgG and IgG2a, NF-ATc1^+^, nitrotyrosine^+^, LRP4^+^, and TCF3^+^ cells in inflamed joints; reduced IL-17 expression and Th17 cells; and increased CD4^+^CD25^+^Foxp3^+^, CTLA-4^+^, PD-1^+^, and GITR^+^ Tregs in the spleen (182, 183). Bone marrow cells from CIA mice were treated with IL-27-Fc in the presence of M-CSF and RANKL, showing less osteoclast differentiation (183). Furthermore, adenovirus containing IL-27 transcript (AdIL-27) was injected into ankles after CIA induction, by which the mice revealed reduced joint circumference and less inflammation, synovial lining thickness, and bone erosion (185). IL-17, hemoglobin, IL-1β, and IL-6 expression in serum was downregulated and CXCL1, CXCL5, and CCL2 expression in joints was reduced in AdIL-27-treated CIA mice (185). In arthritis-susceptible Lewis rats that were injected with IL-27, there was reduced expression of IL-17, RORγt, IL-23, and IL-6 in arthritic joints, and the joint space was clear with minimal MNC infiltration of the synovial tissue (187). The addition of IL-27 to FLSs reduced the activity of MMP-9 and VEGF secretion (187). IL-27rα^−/−^ mice treated with mBSA and complete Freund’s adjuvant showed exacerbated joint pathology, evidenced by elevation of leukocyte infiltration, synovial exudate, hypertrophy, cartilage and bone erosion, elevated expression of IL-17 and mBSA-specific IgG, and increased Th17 cells in dLNs (128). There were increased CD3^+^ and B220^+^ cell infiltration throughout the synovium in IL-27rα^−/−^ mice (128). Thus, IL-27 suppresses osteoclastogenesis and inflammatory cytokine production and inhibits arthritis development. As discussed above, IL-27 has both an anti-inflammatory and pro-inflammatory role in arthritis. This may be attributed to several reasons, mainly including method, time (acute or chronic phase), and duration of IL-27 administration. For example, IL-27rα^−/−^ mice were protected against arthritis (188), whereas the addition of IL-27 to collagen- and osteocollagenesis-induced arthritis may inhibit arthritis development (183). In addition, short-term administration of IL-27 to CIA mice at the onset of the disease will downregulate disease severity (181) and progression (185), whereas IL-27 was not effective in the stable phase. Moreover, the addition of IL-27 at the onset of adjuvant-induced arthritis was able to inhibit joint inflammation; however, if IL-27 was added in a later phase, it will promote the inflammation process (177).

### 4.3 Expression of IL-27 was both increased and reduced in Sjogren syndrome and has a pleiotropic role in Sjogren syndrome pathogenesis

Sjogren syndrome (SS) is an autoimmune disease, and lymphocytic infiltration of the exocrine glands and other organs are the main clinical features of the disorder. In patients with SS from a Northern Chinese Han population and UK, serum levels of IL-27 were elevated as compared to that in healthy controls, and were negatively related to diffusing capacity of the lung for carbon monoxide (%DLCO) (129, 130). Serum levels of IL-27 in patients with SS with or without interstitial lung disease (ILD) were both higher than that in healthy controls, and serum levels of IL-27 were higher in patients with SS with ILD than that in patients with SS without ILD (129) (**Table 1**). In salivary glands (SGs) of patients with SS from UK, there was elevated expression of IL-27p28, Ebi3, and IL-27rα when compared to controls, and IL-27p28 colocalized with DC-LAMP^+^ mature DCs (heavily enriched within the T cell-rich areas of the lymphoid aggregate). Elevated expression of IL-27p28 was also obtained in CD11b^+^ myeloid cells from SGs (130). By contrast, another two studies discussed expression of IL-27 from a Northern Chinese Han population, non-obese diabetic (NOD) mice, showing that levels of IL-27, IL-27rα, and gp130 in serum, plasma, and PBMCs from patients with SS and NOD mice were reduced as compared to those in controls, and were negatively related to expression of IgG (131, 132). Serum levels of IL-27 in patients with active SS, and patients with SSA^+^ and SSB^+^ were much lower as compared to those in patients with inactive SS, and patients with SSA^−^ and SSB^−^. Patients with SS had a lower frequency of Treg cells, a higher frequency of Th17 cells, and increased ratio of Th17/Treg cells, and serum levels of IL-27 were positively related to frequency of Treg cells and negatively related to frequency of Th17 cells (131). Considering the differences in IL-27 expression, this may be attributed to several reasons. For instance, increased serum IL-27 was revealed in the Northern Chinese Han SS population, especially the patients with ILD. It is known that endothelial cells secrete IL-27 (189). Patients with SS with ILD had severe pathogenic changes in the lung, such as greater lymphocyte infiltration, which may trigger increased expression of IL-27. As shown in the studies that found reduced expression of IL-27, patients with SS and mouse models did not show complication of ILD. Moreover, the high expression of IL-27 in SGs from patients with SS showed that DCs are a key source of IL-27, and in this study, the authors did not find higher systemic expression of IL-27 in patients with SS (130). Therefore, the increased expression of IL-27 may be locally produced in patients with SS, and was mainly produced by DCs.

Serum levels of IL-17 were elevated in patients with SS when compared with that in controls, and PBMCs treated with IL-27 showed reduced percentage of CD4^+^IL-17A^+^ T cells, downregulated expression of IL-17A, and increased percentage of Treg cells (130, 131). Naive CD4^+^ T cells from patients with SS were treated under Th17- or Treg-polarizing conditions, and administration of IL-27 suppressed the differentiation of Th17 cells and increased the percentage of Treg cells (131). NOD mice were injected with the serotype 2 adeno-associated viral vector (AAV2)-IL-27, showing reduced IL-17 levels, decreased number of IL-17A^+^ T cells, but increased number of CD3^+^CD4^+^IL-27^+^ cells and IFNγ^+^ cells (190). Lacrimal glands of the AAV2-IL-27-treated NOD mice revealed reduced number of lymphocytic focus (LF)/B cells, and a decrease of serum ANA (190). When NOD mice were injected with recombinant IL-27, there were reduced lymphocytes in SGs/lacrimal glands (LGs), increased salivary flow rate, and upregulated percentage of CD4^+^IL-10^+^ T cells (132). This was confirmed in IL-27^−/−^ NOD mice, where the mice had rash, swollen SGs, and severe inflammation in SGs, LGs, and lung, and the salivary flow rate and number of CD4^+^IL-10^+^ T cells were reduced (131, 132). There were more infiltrating lymphocytes and a larger infiltrating area in SGs of IL-27^−/−^ NOD mice and reduced saliva volume, whereas the addition of IL-27 inhibited lymphocyte infiltration, restored the salivary flow rate, and upregulated the number of CD4^+^IL-10^+^ T cells (132). Similarly, ectopic lymphoid structures (ELSs) were abundant and larger in IL-27rα^−/−^ mice, and there was a higher expression of Ltb, CXCL13, CCL19, CXCR5, CCR7, and activation-induced cytidine deaminase (AID); an exaggerated ectopic GC response; and a higher percentage of GL7^+^ GC B cells in the SGs of IL-27rα^−/−^ mice than those in control mice (130). IL-27rα^−/−^ mice also showed high expression of IgG1, ANA, and anti-adenovirus antibodies in the serum; a high percentage of CD4^+^IL-17A^+^ and CD4^+^IL-22^+^ T cells; and a low percentage of CD4^+^IFNγ^+^ T cells in the SGs (130). However, inflammation in SGs and LGs was reduced in 10-week-old IL-27^−/−^ and IL-27rα^−/−^ NOD mice as compared with age-matched WT NOD mice (191). This protection was not obtained in 30-week-old IL-27^−/−^, IL-27rα^−/−^ NOD mice, suggesting that difference of findings in IL-27^−/− and^ IL-27rα^−/−^ mice was related to the observation time when discussing the role of IL-27 and IL-27rα in SS.

### 4.4 Behcet’s disease had dysregulated expression of IL-27, and IL-27 suppressed Th1 and Th17 cell response

Behcet’s disease (BD) is a chronic vasculitis characterized by multisystem damages, including frequent oral and genital ulcerations, arthritis, and skin lesions. Serum levels of IL-27 were lower in Turkish patients with BD (unknown treatment) as compared to that in healthy controls, which were negatively related to disease duration (133) (**Table 1**). In patients with BD from a Southern Chinese Han population (without treatment), expression of IL-27p28 in PBMCs and IL-27 in serum was both reduced as compared to those in controls (134). Similarly, another study in patients with BD from a Southern Chinese Han population (treated with prednisone) showed that serum IL-27 levels were upregulated after cataract surgery in patients with BD, and serum levels of IL-27 were related to aqueous flare values and cell counts (135). In contrast, a study discussed the serum levels of IL-27 from an Iranian population, showing the higher expression of IL-27 in patients with BD than that in healthy controls (136). Differences between the different expression of IL-27 in patients with BD may correlate with treatment, sample size, and distinct ethnicity. Regarding IL-27 gene polymorphisms, frequencies of the rs153109 AA genotype and rs153109 A allele were higher in Iranian patients with BD as compared to those in controls, whereas genotypes and allele frequencies of rs181206 polymorphism were not different between patients with BD and controls (165) (**Table 2**). This was demonstrated in another study evaluating IL-27 gene rs153109 polymorphism in Iranian patients with BD (136). The study found higher frequencies of AG, GG, and AG+GG genotypes, as well as the G allele of rs153109 in Iranian patients with BD compared with controls. Higher frequencies of AG and GG genotypes and G allele were noted in patients with active disease activity, and joint and vascular damage. Interestingly, patients with BD carrying the rs153109 GG genotype had higher expression of IL-27 than the patients carrying the AA genotype (136). Thus, IL-27 gene rs153109 polymorphism was related to BD risk in an Iranian population. When monocytes from patients with BD were cultured with GM-CSF and IL-4 for DC differentiation, the addition of IL-27 downregulated the expression of IL-1β, IL-6, and IL-23, and upregulated IL-10 production (134). CD4^+^ T cells cultured with IL-27-treated DCs inhibited IL-17 and IFNγ generation, and reduced the frequency of IL-17A^+^ and IFNγ^+^ T cells. However, IRF8^−/−^ DCs treated with IL-27 did not show reduced expression of IL-1β, IL-6 and IL-23 (134). Together, levels of IL-27 were dysregulated in patients with BD, which may inhibit Th1 and Th17 cell response.

### 4.5 High expression of IL-27 in inflammatory bowel disease, which either promotes disease pathogenesis or inhibits development of the disease

Inflammatory bowel disease (IBD) is an inflammatory disease characterized by chronic, relapsing inflammation of gastrointestinal tract, which is related to environmental factors, host immune status, and genetic risk. IBD includes two types: ulcerative colitis (UC) and Crohn’s disease (CD). Expression of IL-27 in PBMCs was comparable between patients with UC and CD in remission state and in the flare-up period after treatment with 5-aminosalicylic acid (5ASA), prednisone (Pred), Azathioprine (AZA), and Infliximab (IFX) (137) (**Table 1**). IL-27 expression in colonic biopsies was increased in patients with active UC versus those with inactive UC, and in patients with active CD versus those with inactive CD, and the percentage of IL-27 immunoreactive cells was increased in patients with active UC as compared to that in patients with active CD (138). Expression of IL-27p28 and Ebi3 was increased in colonic mucosa from patients with active CD when compared to that in controls (139). In patients with CD, IL-27rα was highly expressed in infiltrating immune cells from inflamed intestinal mucosa than that in normal intestinal epithelial cells (IECs) (140). The inflamed tissue had elevated expression of gp130 in epithelial cells (140). Moreover, there were increased serum levels of IL-27 in *Clostridium difficile*-induced (CDI) colitis patients as compared to that in healthy controls, and the IL-27 expression in stools from CDI patients was significantly increased (141). Expression of IL-27p28 and Ebi3 was increased in moDCs isolated from CDI patients after *C. difficile* stimulation, and WT mice infected with *C. difficile* showed an increase in serum and cecal IL-27 (141). For IL-27 genetic mutation, rs153109 of IL-27 was related to UC in Northern Chinese Han individuals, and the patients carrying the A allele of rs153109 showed reduced expression of IL-27 in PBMCs (166). The risk A allele in rs153109 was able to inhibit the promoter activity of the *IL-27* gene compared to the G allele (166). The rs153109 polymorphism correlated with UC and CD risk in a Korean population, and haplotype AGT [rs153109 (A) + rsrs17855750 (G) + rs181206 (T)] positively correlated with UC and CD risk, and haplotype GGT [rs153109 (G) + rsrs17855750 (G) + rs181206 (T)] negatively correlated with UC and CD risk (167) (**Table 2**). IL-27p28 rs17855750 polymorphism was negatively related to UC susceptibility, and Ebi3 rs428253, rs4740, and rs4905 polymorphisms were negatively related to UC risk in a Mexican population (168). Collectively, IL-27 expression was increased in IBD patients, and IL-27 gene polymorphisms were related to IBD risk.

IBD patients’ colon-derived organoid monolayer expressed IL-27rα (192). When the organoid monolayer was exposed to TNFα, the barrier was damaged, whereas the addition of IL-27 led to a restitution of barrier integrity, downregulated the expression of claudin-2 and ZO-1, and upregulated the expression of Claudin-4, occludin, and E-cadherin, indicating that IL-27 may improve barrier integrity by inhibiting TNFα-mediated effects (192). When primary colonic epithelial cells isolated from CDI patients were stimulated with IL-27, the expression of Cathelicidin was increased; however, the expression of Cathelicidin was reduced in IL-27-treated cells in the presence of JAK2, PI3K, or p38 inhibitors (193). DLD-1 cells simulated with IL-27 activated the ERK, p38, Akt, STAT1, STAT3, and STAT6 signaling, and increased the expression of alpha-2-macroglobulin (A2M), basic leucine zipper transcriptional factor ATF-like 3 (BATF3), deleted in malignant brain tumor (DMBT1), IDO1, and SOCS3 (140). IL-27 stimulation increased DLD-1 cell proliferation, whereas the addition of p38 inhibitor or silencing STAT6 inhibited cell proliferation. In STAT1^−/−^ DLD-1 cells, stimulation with IL-27 upregulated cell restitution, and IL-27-mediated cell migration was inhibited by silencing STAT3 or STAT6 (140). When human CD4^+^ T cells were treated under Th17-polarizing conditions in the presence of an IL-27 heterodimer scIL-27 (a fusion protein of Ebi3 to p28), scIL-27 suppressed IL-17A production and inhibited the percentage of CD4^+^IL-17A^+^ T cells, but the inhibition was abolished by the addition of anti-IL-27 antibody (194). Therefore, IL-27 may regulate IBD-related cell differentiation and inflammatory factor production by downstream signaling pathways.

It has been reported that the role of IL-27 in IBD pathogenesis was inconsistent. There were reduced frequencies of CD4^+^IFNγ^+^ T cells, higher frequencies of Th17 cells, and increased IL-17A expression in mesenteric lymph nodes (mLNs), intraepithelial lymphocyte (IEL), and lamina propria of the colon (LPL) isolated from dextran sodium sulfate (DSS)-treated IL-27rα^−/−^ mice (195). The ratio of Th17/Th1 cells after DSS treatment was elevated in IL-27rα^−/−^ mice. DSS-treated IL-27rα^−/−^ mice had severe intestinal inflammation (evidenced by loss of crypt architecture, epithelial damage, numerous macrophages, and neutrophils in lamina propria), lost much weight, and displayed diarrhea, intestinal bleeding, and increased morbidity (195). When cotransferring naive T cells from IL-27rα^−/−^ mice with B cells from WT mice into B6 DKO mice, there was severe colitis, and IFNγ and IL-17A expression in colonic tissue explants was increased and IL-10 expression was reduced (196). IL-27rα^−/−^ mice infected with *C. difficile* had reduced expression of CRAMP in the colonic tissues, stools, and serum when compared to that in control mice (193). IL-27rα^−/−^ mice infected with *C. difficile* also reported more weight loss; higher expression of IL-6, IL-17A, and IL-23 in cecal tissue; lower IFNγ and IL-10 expression in cecal tissue; lower survival rate; and shorter colon length than WT mice (141). Interestingly, IL-27rα^−/−^ mice treated with AOM and DSS had upregulated tumor load, tumor numbers, and tumor size, and increased percentage of myeloid-derived suppressor cells (MDSCs) (197). Moreover, 2,4,6-trinitrobenzenesulfonic acid (TNBS)-treated WT mice showed acute, severe, left-sided colitis, whereas delivering *Lactococcus lactis*-expressing IL-27 (LL-IL-27) inhibited TNBS-induced colitis and weight loss (198, 199). LL-IL-27 delivery in TNBS-induced colitis mice also led to increased colon length; decreased serum levels of C-reactive protein (CRP), CXCL1, CXCL2, IL-1α, IL-6, IFNγ, IL-23, IL-17A, and IL-17F; reduced number of IL-17A- and IL-17F-expressing cells; reduced total histology colitis score; and myeloperoxidase^+^ neutrophil infiltration into the colonic mucosa (198, 199). The role of scIL-27 was confirmed in TNBS-induced colitis mice and DSS-induced colitis mice (194), by which scIL-27-treated colitis mice revealed reduced severity of colitis, evidenced by recovery of body weight; reduced DAI score; improved colon length; reduction in macroscopic scores; reduced expression of IL-1β, IL-6, TNFα, IL-10, IFNγ, and IL-17A; less severity of transmural inflammation characterized by ulceration; loss of goblet cells; and tissue disruption throughout the colon. In AAV-IL-27-treated colitis mice, there were no signs of wasting diseases, such as loss of body weight, hunched-over appearance, piloerection of the coat, diarrhea, and blood in the stool; colon from the AAV-IL-27-treated mice revealed no significant histopathological changes (200). Furthermore, IL-10^−/−^ mice infected with *Citrobacter rodentium* showed intestinal inflammation, and administration of neutralizing anti-IL-27 antibody had significant mucosal infiltration with inflammatory cells and extensive submucosal swelling, along with elevated expression of IL-6 (201). Therefore, the above findings demonstrated that IL-27 inhibits colitis pathogenesis. In contrast, DSS-treated IL-27rα^−/−^ mice showed mild body weight loss and less hematochezia, lower histological scores, and longer colonic length as compared to control mice, and there were less inflammation and crypt damage in the colons (202). Lamina propria mononuclear cells (LPMCs) and mLN cells from DSS-treated IL-27rα^−/−^ mice produced lower expression of IFNγ, IL-6, TNFα, and T-bet, and serum levels of IL-6 and TNFα were lower than those in control mice (202). Transferring IL-27rα^−/−^ CD45Rb^hi^ T cells into CB17-SCID recipient mice led to protection against weight loss, and showed decreased colonic shortening and histological scores (203). Transferring WT CD4^+^ T cells into TCRβ^−/−^ mice expressing IL-27rα led to body weight loss, heavily infiltrated with inflammatory cells (CD3^+^ T cells and macrophages), whereas IL-27rα^−/−^TCRβ^−/−^ mice that received WT CD4^+^ T cells displayed no signs of weight loss, and the colon architecture and goblet cells remained intact with minor inflammation, and the percentage of CD4^+^IL-17A^+^ T cells and IL-1β and IL-6 expression were reduced (204). Interestingly, IL-10^−/−^ mice had signs of intestinal disease, including weight loss; prolapsed rectum; increased expression of IL-12p35, IL-23p19, IL-17A, IL-17F, IL-22, IFNγ, IL-27p28, and Ebi3; severe crypt elongation and hyperplasia of mononuclear cell infiltrates; and disruption of epithelial barrier in the colonic section (205). However, IL-10^−/−^IL-27rα^−/−^ mice had mild crypt elongation and reduced expression of the pro-inflammatory cytokines, suggesting that IL-27 may promote colitis development (205). Thus, IL-27 may regulate IL-10-induced colitis. With respect to the differences in the role of IL-27 in colitis, the reasons may correlate with following: For instance, it is unclear that IL-27 plays a pro-inflammatory or an anti-inflammatory role in different immune cells, and then contributes to colitis or inhibits colitis. This may relate to stimulus, cell type, and the surrounding/experimental microenvironment (206). To date, there is growing evidence suggesting that Th17-related cytokines contribute to colitis (207). All the findings discussed the role of LL-IL-27, AAV-IL-27, scIL-27, and anti-IL-27 antibodies in colitis and demonstrated that IL-27 inhibits colitis by suppressing Th17-mediated immune responses (194, 198–201). B6 DKO mice cotransferred T cells from IL-27rα^−/−^ mice and B cells from WT mice showed signs of colitis, whereas B6 DKO mice cotransferred T cells from IL-27rα^−/−^ mice and B cells from IL-10^−/−^ mice had no features of colitis, demonstrating the role of IL-27 signaling in regulating IL-10-secreting B cells to ameliorate T cell-mediated colitis and contribute to intestinal homeostasis (196). For IL-27 promoting colitis, a study found that IL-27-mediated stimulation of non-T cells (especially macrophages and DCs) was a risk for intestinal inflammation (204). The study that transferred IL-27rα^−/−^CD45Rb^hi^ T cells into CB17-SCID mice found inhibited colitis (203). The finding was related to the IL-27rα^−/−^CD45Rb^hi^ T cells, which assumed a Foxp3^+^ phenotype, demonstrating that IL-27 suppressed the Treg-like cells and then displayed the pro-inflammatory mechanism (203).

### 4.6 Multiple sclerosis displayed low expression of IL-27, along with the negative role of IL-27 in multiple sclerosis pathogenesis

Multiple sclerosis (MS) is a chronic inflammatory and autoimmune demyelinating disease of the central nervous system (CNS). Several studies evaluated serum and plasma levels of IL-27 in patients with MS without treatment and healthy controls, showing that patients with MS had lower expression of IL-27 (142–145) (**Table 1**). There were higher plasma IL-17 levels and frequencies of peripheral blood Th17 cells in patients with MS, and plasma levels of IL-27 were negatively related to IL-17 expression and the frequency of Th17 cells in the patients (143). In contrast, some other studies found higher expression of IL-27 in patients with MS after treatment (145, 146). Cerebrospinal fluid (CSF) and serum from patients with MS under disease-modifying treatment both showed higher expression of IL-27 as compared to that in controls (145). Expression of IL-27 was related to white blood cell (WBC) counts in the CSF of patients with MS, and CSF levels of IL-27 were lower in patients with MS who did not present oligoclonal IgG bands (OB) positivity. Ebi3 was highly expressed at the rim of the demyelinated lesion (145). Moreover, several studies also found elevated expression of IL-27 in patients with MS that did not show whether there is treatment in patients with MS and EAE mice (147–149). In our opinion, it is hypothesized that patients with MS without treatment may reveal a relatively low expression of IL-27, whereas treatment in patients with MS may upregulate the expression of IL-27. Furthermore, all the studies that evaluated the expression of IL-27 in patients with MS had a small sample size, and detecting methods were partly different. Thus, in the future, larger samples and the same method need to confirm the real expression profile of IL-27 in MS. The IL-27 rs181206 polymorphism was related to MS risk in Romania, by which the frequencies of TC+CC genotypes were higher in patients with MS as compared to that in healthy controls (142, 169). Patients with MS carrying IL-27 rs153109 AG+GG genotypes also showed higher risk of MS compared to non-carriers, and patients carrying the GC haplotype [rs153106 (G) + rs181206 (C)] reported a higher risk of developing MS (169) (**Table 2**). This may suggest that IL-27 gene polymorphisms (rs181206 and rs153109) were related to MS risk in Romania.

In patients with MS, there were more CD4^+^ T cells that produced IL-17A, GM-CSF, and IFNγ than those from healthy controls, and the percentage of STAT3^+^ cells in CD4^+^ and CD8^+^ T cells from patients with MS was increased (169). The addition of IL-27 to CD4^+^ T cells from patients with MS downregulated the percentage of CD4^+^IL-17A^+^ and CD4^+^GM-CSF^+^ T cells, reduced the expression of IL-17A, but activated STAT3 signaling (169). Human moDCs stimulated with zymosan had elevated production of IL-1β and IL-23, whereas the addition of IL-27 suppressed zymosan-induced IL-1β and IL-23 expression (208). Coculturing zymosan-treated moDCs with allogeneic CD4^+^ T cells in the presence of IL-27 showed low levels of IL-17 and increased the expression of IL-10. Similarly, zymosan-treated moDCs in the presence of INFβ had reduced expression of IL-23 and IL-1β; however, the addition of neutralizing antibody for IL-27 reversed the expression of IL-23 and IL-1β, suggesting that IL-27 regulates TLR-induced cytokine production through DCs and mediates the inhibitive effects of IFNβ on Th17 cells (208). Human astrocytes treated with IL-27 showed increased percentage of astrocytes expressing MHC I; however, the elevated expression was abolished when the astrocytes were pretreated with STAT1, indicating that IL-27 shapes astrocyte immune function, for instance, MHC I expression in a STAT1-dependent manner (149). WT mice immunized with MOG_35-55_ showed severe clinical features of MS, evidenced by significant inflammation and demyelination in spinal cords, whereas the addition of IL-27 delayed the onset of the disease, downregulated disease severity, and upregulated recovery (209). In both dLNs and splenocytes from IL-27-treated EAE mice, there was reduced expression of IL-17 and IFNγ (209). Furthermore, injection of AAV-IL-27 into EAE mice prevented EAE development; downregulated the expression of GM-CSF, IL-17, and Foxp3; and upregulated the expression of IL-10 and IFNγ in CD4^+^ T cells from lymph nodes and spleen (210). The percentage of CD11b^+^Gr1^+^ myeloid cells was elevated in spleen and CNS of EAE mice, and injection of AAV-IL-27 further expanded these cells (210). All these demonstrated that IL-27 suppresses the pathogenesis of MS.

### 4.7 Dual role of IL-27 in psoriasis pathogenesis

Psoriasis is a systemic, immune-mediated disease that mostly involves skin inflammation and joint damage. Serum levels of IL-27 were elevated in patients with psoriasis compared to those in healthy controls, and serum levels of IL-27 were related to psoriasis area, severity index (PASI) score, and serum levels of IFNγ (150, 151) (**Table 1**). IL-27^+^ cells were obtained in the papillary dermis in psoriatic skin lesions (151). However, another study showed that IL-27^+^ skin cells were reduced in the psoriatic lesions, and there was lower expression of IL-27rα, IL-27p28, and gp130 in epidermis and dermis from patients with psoriasis (152). Expression of IL-27p28 was reduced in PBMCs of patients with psoriasis compared with healthy controls, and serum levels of IL-27 were reduced in patients with psoriasis (152). Different findings may relate to treatment, sample size, and detecting method. The study evaluated the serum levels of IL-27 in patients with psoriasis who were treated with steroids and/or vitamin D3 (151). The above three studies recruited a relatively small sample of patients with psoriasis (150–152). Detecting methods were different among the three studies, especially the two studies that purchased enzyme-linked immunosorbent assay kits from different companies and showed different serum levels of IL-27 (150, 151).

Human primary dermal fibroblasts exposed to IL-27 upregulated IL-18BP expression and activated STAT1 (211). IL-27 binds to the IL-18BP WT promoter, which leads to the activation of the promoter, whereas IL-27 binds to the mutated proximal GAS site in the IL-18BP promoter, which results in the reduced activation of the IL-18BP promoter, suggesting that the GAS site was important for gene activation in response to IL-27 (211). Human keratinocytes exposed to IL-27 increased the expression of CXCL9, CXCL10, and CXCL11, and upregulated the phosphorylation expression of STAT1 (151). WT mice treated with imiquimod (IMQ) displayed phenotypes of psoriasis, such as severe erythema, thickness, scales, and high expression of IL-17 (152). The administration of IL-27 into psoriasis mice reduced disease severity, showing less disease score and less acanthosis and dermal infiltrate of inflammatory cells, whereas the addition of anti-IL-27p28 antibody to psoriasis mice exacerbated skin inflammation (152). Injection of IL-27 into psoriasis mice upregulated serum levels of IFNγ and decreased serum levels of IL-17, but inhibition of IL-27 downregulated the expression of IFNγ and increased IL-17 expression. CD4^+^ T cells from spleen of psoriasis mice stimulated with IL-27 showed reduced expression of IL-17, whereas anti-IL-27p28 antibody resulted in the elevated expression of IL-17 (152). By contrast, another study discussed the role of IL-27 in IMQ-induced psoriasis mice, showing worse clinical outcome, evidenced by severe scales, thicker skin, and more epidermal hyperplasia with elongation of rete ridges, and inflammatory cells infiltrate in IL-27 injected mice (212). Injection of anti-IL-27p28 antibody into IMQ-induced psoriasis mice improved disease severity. IL-27-treated mice also showed elevated expression of IFNγ, but anti-IL-27p28 antibody injection downregulated the expression of IFNγ (212). Differences in the role of IL-27 in IMQ-induced psoriasis mice may be attributed to several reasons. First, the finding of IL-27-inhibited psoriasis may correlate with the suppressive role of IL-27 in Th17 cells. As is known, Th17 cells and related cytokines are involved in psoriasis pathogenesis. The study showed that IL-27 injection inhibited the expression of IL-17 in serum, skin, and CD4^+^ T cells. Another study found that the pro-inflammatory role of IL-27 may be correlated with the role of IL-27 in IFNγ, by which the injection of IL-27 promoted the expression of IFNγ. Therefore, IL-27 may activate Th1-mediated response in psoriasis, such as regulating the expression of IFNγ, and may be involved in psoriasis development. Moreover, the two studies selected different drug-delivery methods: one treated the mice with a continuous pumping of IL-27, and the other one administrated IL-27 by intermittent subcutaneous injection; thus, the time-dependent changes in drug concentrations may result in distinct outcomes (152, 212).

### 4.8 IL-27 has different roles in the pathogenesis of systemic sclerosis

Systemic sclerosis (SSc) is an autoimmune connective tissue disease characterized by fibrosis, diffuse vasculopathy, and inflammation. The serum levels of IL-27 were higher in patients with SSc that that in healthy controls (153) (**Table 1**). The serum levels of IL-27 in patients with diffuse cutaneous SSc (dSSc) and patients with limited cutaneous SSc (lSSc) were increased as compared to that in healthy controls. The serum levels of IL-27 were higher in patients with dSSc as compared to that in lSSc. When dividing patients with SSc into two groups, patients with elevated expression of IL-27 and patients with normal expression of IL-27, longer disease duration, lower levels of IgG, and modified Rodnan TSS points were obtained in patients with normal expression of IL-27. There were increased %vital capacity (VC), %diffusion capacity for carbon monoxide, reduced levels of hyaluronan, and less pulmonary fibrosis in patients with normal expression of IL-27 (153). The serum levels of IL-27 were related to levels of IgG and IL-17. The expression of IL-27rα in B cells and CD4^+^ T cells from patients with dSSc was higher than that in healthy controls. B cells from patients with dSSc treated with hyaluronan and IL-27 upregulated the expression of IgG and IL-17. Stimulation of dSSc fibroblasts with IL-17 upregulated the expression of IL-27rα, and stimulation of fibroblasts with IL-17 and IL-27 further upregulated the expression of IL-27rα, type I collagen (COL1A1), and COL1A2, revealing that IL-27 may interact with IL-17 and then upregulate inflammatory component generation (153). In bleomycin (BLM)-induced pulmonary fibrosis mice, there were thickened pulmonary interalveolar septa and inflammatory cells infiltrate (213). The addition of anti-IL-27 antibody exacerbated the disease severity, downregulated survival, and upregulated mortality rate and expression of hydroxyproline and type I and III collagen, whereas injection of IL-27 relieved disease activity and degree of alveolitis (213). There were elevated percentages of CD4^+^IL-4^+^, CD4^+^IL-10^+^, and CD4^+^IL-17^+^ T cells; increased expression of IL-4, IL-10, IL-17, and TGF-β; decreased percentages of CD4^+^IFNγ^+^ and CD4^+^Foxp3^+^ T cells; and reduced expression of IFNγ in anti-IL-27 antibody-treated mice, whereas the addition of IL-27 significantly reversed anti-IL-27 antibody-induced effects (213). Interestingly, stimulation of lung fibroblasts (LFs) with TGF-β1 increased proliferation and viability of the cells, and upregulated the expression of MMP-2 and MMP-9, but treatment with IL-27 decreased proliferation and viability of the cells, and suppressed the expression of alpha-smooth muscle actin (α-SMA), tissue inhibitor of metalloproteinase-1 (TIMP-1), and type I and III collagen (214). LFs stimulated with TGF-β1 activated JAK1, STAT1, STAT3, and STAT5, whereas IL-27 stimulation inhibited phosphorylation of the signaling. It is known that proliferation and collagen synthesis of the LFs were inhibited under TGF-β1 stimulation in the presence of JAK/STAT signaling inhibitor, suggesting that IL-27 performs an anti-fibrotic role in LFs. As discussed above, the findings for IL-27 in SSc were different. This may correlate with the consequence of IL-27 signaling, which depends on the disease model, time point, and immunological context. For instance, the two studies found an inhibitive role of IL-27 in fibrosis, and BLM-induced pulmonary fibrosis mice may correlate with the effects of IL-27 on inhibiting Th2 and Th17 cells and related inflammatory cytokines and promoting the differentiation of Treg cells. In addition, the pro-inflammatory role of IL-27 was reported in patients with SSc, whereas the two studies found an inhibitive role of IL-27 in fibrosis, which was discussed in animal models. Therefore, further studies in both patients and animal models are still needed.

### 4.9 Pro-inflammatory and anti-inflammatory effects of IL-27 in type 1 diabetes

Type 1 diabetes (T1D) is a chronic autoimmune disease in which islet β cells are destroyed and is characterized by the lack of insulin. A study that evaluated the serum levels of IL-27 found that they were not different between patients with T1D and healthy controls (154) (**Table 1**). However, concentrations of IL-27 were positively related to sirtuin-1 expression and thyroid volume, and negatively related to relative wall thickness (154). Another study detected IL-27 expression profile in serum, showing that serum levels of IL-27 in patients with T1D, patients with T1D with normal HbA1c, or patients with T1D with high HbA1c were lower than that in healthy controls (155). There was a higher ratio of IL-17/IL-27 in patients with T1D than that in healthy controls (155). By contrast, the plasma levels of IL-27 were reported to be elevated in patients with T1D compared to healthy controls, and were related to the plasma levels of IL-17A, IL-21, IL-22, and IL-23 in patients with T1D (156). In Treg cells from patients with T1D, the expression of IL-27 was lower than that in controls (157). The findings suggest that the expression of IL-27 was abnormal in patients with T1D. Regarding the serum/plasma levels of IL-27, the three studies included patients with T1D either with treatment (treatment of insulin) or without treatment; therefore, results were different (154–156). Second, all the studies had a small sample size. Third, different methods to detect the expression of IL-27 may result in distinct results. IL-27 gene polymorphisms (rs153109, rs34833, rs26528, rs17855750, rs181206, and rs40837) were not related to T1D risk in Brazil, and IL-27 genetic mutation did not correlate with gender, age at diagnosis of diabetes, and patients with pancreatic and extrapancreatic autoantibodies (170) (**Table 2**).

CD44^high^SLAMF6^+^CXCR6^−^ cells and CD44^low^SLAMF6^+^CXCR6^−^ cells from NOD mice were simulated with IL-27, showing increased percentage of CD44^high^SLAMF6^+^CXCR6^−^ cells with the TCF1^−^CXCR6^+^ phenotype, and elevated proportion of CD44^low^SLAMF6^+^CXCR6^−^ cells with the TCF1^−^CXCR6^+^ phenotype, respectively, suggesting the role of IL-27 in promoting the generation of CD44^high^TCF1^−^CXCR6^+^ T cells from CD44^high^TCF1^+^CXCR6^−^ T cells (88). WT mice were treated with streptozotocin, and IL-23 induced diabetes, evidenced by hyperglycemia, weight loss, and mononuclear cell infiltration. The addition of IL-27 reduced the incidence of diabetes and disease severity (215). Therefore, IL-27 limited IL-23-induced diabetes. Ebi3^−/−^ and IL-27rα^−/−^ mice treated with streptozotocin reported elevation of intraislet and peri-islet mononuclear cell infiltration, lower expression of cellular proinsulin, and a higher number of polymorphonuclear leukocytes, CD11c^−^ cells, CD4^+^ cells, and CD8^+^ cells in islets as compared to those in WT mice (216). In contrast, anti-IL-27 antibody-treated diabetic splenocytes were transferred into NOD-SCID mice, showing increased incidence of diabetes, elevated blood glucose and IFNγ and IL-17 expression, and reduced expression of IL-4, TGF-β, and IL-10 (217). Both IL-27^−/−^ NOD mice and IL-27rα^−/−^ NOD mice were resistant to T1D development, by which insulitis was reduced (191). Transferring bone marrow (BM) cells from Rag1^−/−^ NOD mice into IL-27^−/−^ NOD mice upregulated the incidence of diabetes. There were low percentages of CD45^+^, CD3^+^, and CD103^+^ migratory DCs in islets of IL-27^−/−^ NOD mice. When T cells from NOD or IL-27rα^−/−^ NOD mice were transferred into Rag1^−/−^ NOD recipients, the mice that received T cells from IL-27rα^−/−^ NOD mice did not induce T1D. Interestingly, CD4^+^ and CD8^+^ T cells from NOD mice were transferred into Rag1^−/−^ NOD recipients, or CD4^+^ and CD8^+^ T cells from IL-27rα^−/−^ NOD mice were transferred into Rag1^−/−^ NOD recipients. Co-transfer of CD4^+^ and CD8^+^ T cells from NOD mice into Rag1^−/−^ NOD mice upregulated the incidence of diabetes, whereas co-transfer of CD4^+^ and CD8^+^ T cells from IL-27rα^−/−^ NOD mice into Rag1^−/−^ NOD mice did not induce diabetes, demonstrating that IL-27 signaling is essential for T1D development (191). As discussed above, studies that used NOD mice as a disease model showed a pro-inflammatory role of IL-27 in diabetes development, but studies that selected streptozotocin to induce diabetes showed an anti-inflammatory role of IL-27 in diabetes pathogenesis. This may contribute to different results of IL-27 in diabetes.

### 4.10 Pleiotropic features of IL-27 in uveitis pathogenesis

Autoimmune uveitis is an inflammatory autoimmune disease, and Vogt–Koyanagi–Harada (VKH) disease is one of the most common uveitis entities. Patients with active VKH had reduced expression of IL-27p28 in PBMCs and lower serum levels of IL-27 as compared to those in healthy controls (158) (**Table 1**). After surgery, patients with VKH showed elevated serum levels of IL-27 (159). Interestingly, there was no significant association of IL-27 rs4788084 polymorphism and anterior uveitis risk in Chinese patients (171). Stimulation of CD4^+^ T cells from patients with VKH with Th17-polarizing condition revealed high Th17 cell differentiation, and the addition of IL-27 suppressed Th17 cell differentiation. When moDCs from patients with VKH were cocultured with CD4^+^ T cells in the presence of IL-27, there was less Th17 cell differentiation (158). Retina cells from WT mice or STAT1^−/−^ mice were stimulated with IL-27, showing the increased expression of IL-10 in IL-27-treated cells from WT mice but not in STAT1^−/−^ mice, suggesting that IL-27 may promote the production of IL-10 by activating the STAT1 pathway (218). WT mice immunized with interphotoreceptor retinoid-binding protein (IRBP) developed experimental autoimmune uveitis (EAU), evidenced by severe inflammation with papilledema, retinal vasculitis, and choroidal infiltrate (219). IL-27 injection downregulated T-cell proliferation and expression of IL-2 and IL-17 and upregulated the expression of SOCS3 from lymph node in EAU mice (220). Similarly, injection of IL-27p28 decreased disease severity of EAU and EAU scores, downregulated percentages of Th1 and Th17 cells, and upregulated the proportion of Foxp3^+^ and IL-10-expressing Tregs in spleen (219). T cells from EAU mice stimulated with IRBP in the presence of IL-27p28 revealed reduced proliferation of T cells. Transferring T cells from EAU mice that were stimulated with IL-27p28 and IRBP into unimmunized mice did not induce uveitis (219). IL-27p28 transgenic mice immunized with IRBP did not develop significant uveitis, showing low disease scores, few percentages of CD4^+^IL-17A^+^ and CD4^+^IFNγ^+^ T cells in the eyes, and reduced CD4^+^GM-CSF^+^ T cells in the ocular cell infiltrate. When CD4^+^CD62L^+^ T cells from IL-27p28 transgenic mice were treated under Th1-polarizing conditions, IFNγ generation was downregulated, and the expression of TBX21 and IL-12Rβ2 and the phosphorylation expression of STAT1 and STAT3 were inhibited. When CD4^+^CD62L^+^ T cells from IL-27p28 transgenic mice were treated under Th17-polarizing conditions, the expression of IL-17A was suppressed, along with the decreased expression of IL-23R and RORγt (221). The findings were confirmed in IL-27rα^−/−^ mice, where IL-27Rα^−/−^ mice immunized with IRBP revealed higher EAU scores, severe vasculitis/retinitis and perivascular exudates, lower electroretinogram amplitudes, and greater CD4^+^IFNγ^+^, CD4^+^IL-17A^+^, CD4^+^GM-CSF^+^, CD4^+^IFNγ^+^GM-CSF^+^, and CD4^+^IL-17A^+^GM-CSF^+^ T-cell infiltration in the eyes. There was a higher expression of IFNγ and IL-17A in CD4^+^ T cells from spleen in IL-27rα^−/−^ EAU mice (222). However, another study discussed IL-27rα^−/−^ mice immunized with IRBP, showing lower EAU scores, and the margins of the optic discs were clear, with only minimal pathological changes and reduced expression of IFNγ, RANTES, IP-10, and MCP-1 in the eyes as compared to those in control mice on day 19 after induction. It is notable that clinical scores were similar between IL-27rα^−/−^ EAU mice and control mice on day 21 (223), suggesting that the difference of IL-27rα in EAU development depends on observation time.

### 4.11 Elevated expression of IL-27 in ankylosing spondylitis, and IL-27 limited autoimmune gastritis development

Ankylosing spondylitis (AS) is a seronegative inflammatory rheumatic disease characterized by an imbalance of the immune system and inflammation in the joints. Serum levels of IL-27 were much higher in patients with AS as compared to that in healthy controls, and were related to serum levels of VEGF and Bath Ankylosing Spondylitis Disease Activity Index (BASDAI) (160) (**Table 1**). Patients with AS with peripheral arthritis or positive HLA-B27 showed higher serum levels of IL-27 and VEGF as compared to the patients without such features. The SF levels of IL-27 in patients with AS with peripheral arthritis were higher than the serum levels of IL-27 in patients with AS with peripheral arthritis, indicating that IL-27 may participate in the inflammatory process of AS (160). TxA23xEbi3^−/−^ mice develop autoimmune gastritis but do not express IL-27 (224). Stomachs from TxA23xEbi3^−/−^ mice showed severe disease activity, such as inflammation, parietal cell atrophy, mucinous hyperplasia/metaplasia, and mucosal hyperplasia. When IL-27 was injected into TxA23xEbi3^−/−^ mice, stomachs were much healthier, evidenced by the reduction in the extent of atrophy, mucinous hyperplasia/metaplasia, and inflammatory infiltrate, and there were lower scores for inflammation, atrophy, and mucinous hyperplasia/metaplasia, demonstrating that IL-27 is able to inhibit autoimmune gastritis (224).

## 5 Discussion

This study systematically reviewed and discussed the association of bifunctional IL-27 in inflammatory autoimmune diseases according to the role of IL-27 in different immune cells. This updated advancement of bifunctional IL-27 in autoimmune disorders is summarized in both human and animal model studies. All this information showed that IL-27 has played significant roles in autoimmunity. However, several points need to be further discussed in the future. First, it is accepted that genetic mutation may affect mRNA expression of a gene, which will then change protein expression (225, 226). Therefore, IL-27 gene polymorphisms may indeed change the mRNA and protein expression of IL-27, which is involved in the pathogenesis of different autoimmune diseases. For example, the rs153109 polymorphism was not related to SLE risk in Egyptian and Polish patients, but the rs17855750 polymorphism was related to SLE risk in Egyptian patients. Therefore, more SNPs of the IL-27 gene and more ethnicities and samples are still necessary to confirm the association of IL-27 genetic mutation with inflammatory autoimmune disorders. Similarly, how mutated polymorphism regulated the expression of IL-27 and then promoted disease development is warranted. Based on this circumstance, targeting the mutated IL-27 polymorphism may help to monitor treatment effectiveness in patients. Second, in this study, we have clearly revealed when and where IL-27 plays an anti-inflammatory role in certain immune cells or performs pro-inflammatory activity on certain immune cells. The different roles of IL-27 are mainly attributed to the phase of the disease, stimulation time, and cell type under study. Thus, more studies with clinical trials involving patients with various diseases need to be conducted in the future. When designing a clinical study, the focus should be on the effect of IL-27 on certain immune cells, and changes in disease activity, downstream signaling, and cytokine expression should be evaluated. It is hoped that this precision treatment in the future will be more effective and safer for patients. For instance, methods such as mAb to inhibit highly expressed IL-27 in CD14^+^ monocytes in patients with RA may reverse severe RA disease and suppress highly expressed pro-inflammatory components in circulation and local joints.

## Author contributions

W-DX: Writing – original draft, Writing – review & editing. D-CW: Writing – original draft, Writing – review & editing. MZ: Writing – original draft, Writing – review & editing. A-FH: Writing – original draft, Writing – review & editing.
